# Increasing the Lifetime of Mobile WSNs via Dynamic Optimization of Sensor Node Communication Activity

**DOI:** 10.3390/s16091536

**Published:** 2016-09-20

**Authors:** Dayan Adionel Guimarães, Lucas Jun Sakai, Antonio Marcos Alberti, Rausley Adriano Amaral de Souza

**Affiliations:** National Institute of Telecommunications—Inatel Av. João de Camargo 510, Santa Rita do Sapucaí, MG 37540-000, Brazil; lucas.sakai@inatel.br (L.J.S.); alberti@inatel.br (A.M.A.); rausley@inatel.br (R.A.A.d.S.)

**Keywords:** convex optimization, energy harvesting, greedy algorithm, lifetime optimization, mobile wireless sensor networks, wireless power transmission

## Abstract

In this paper, a simple and flexible method for increasing the lifetime of fixed or mobile wireless sensor networks is proposed. Based on past residual energy information reported by the sensor nodes, the sink node or another central node dynamically optimizes the communication activity levels of the sensor nodes to save energy without sacrificing the data throughput. The activity levels are defined to represent portions of time or time-frequency slots in a frame, during which the sensor nodes are scheduled to communicate with the sink node to report sensory measurements. Besides node mobility, it is considered that sensors’ batteries may be recharged via a wireless power transmission or equivalent energy harvesting scheme, bringing to the optimization problem an even more dynamic character. We report large increased lifetimes over the non-optimized network and comparable or even larger lifetime improvements with respect to an idealized greedy algorithm that uses both the real-time channel state and the residual energy information.

## 1. Introduction

The presence of mobile sink nodes, mobile sensors or both in a wireless sensor network (WSN) characterizes a mobile wireless sensor network (MWSN) [1,2]. An MWSN has a dynamic topology, and for this reason it poses a series of restrictions to the system design, for instance as concerns routing and medium access control (MAC) protocols. Additionally, the communication links may become unreliable as the components of the network move, bringing restrictions to the design of strategies for quality of service (QoS) control. Moreover, determining the optimum location of the sink nodes for maximum energy efficiency becomes a quite involved problem in mobile networks. Nonetheless, mobility brings not only problems and restrictions to the MWSNs. It can be explored to improve coverage, to increase the network lifetime and to handle energy control [2]. Energy management is another important issue to be considered, since most of the sensor networks have their lifetime (or lifespan) increased if the limited energy residual to the sensor nodes can be saved somehow.

The applications for MWSNs include those of traditional WSNs, viz. environmental monitoring, military surveillance, smart homes, health monitoring, manufacturing control, vehicle tracking and other telematics applications. Specific MWSN applications are, for instance [2]: mobile phones acting as sink nodes to gather information from sensors placed anywhere, in applications related to smart transportation systems, security, social interaction, health, wildlife monitoring, and so on. A potentially interesting envisaged application is related to the recent concept of cognitive radio (CR) [3]: a dedicated or multipurpose MWSN can be used for spectrum sensing, and mobile secondary terminals can act as sink nodes to gather information on spectrum availability for subsequent dynamic spectrum access [4]. Additionally, health applications of WSNs are gaining special attention in recent research, for example the emerging wireless body area networks (WBANs) with implanted sensors [5]. In this application, the battery lifetime and, thus, the network lifetime are crucial problems since batteries cannot be replaced without a complex invasive medical procedure. Aiming at maximizing the battery lifetime, non-active sensors can be kept in a sleep state. During this state, a wake-up and charge process can take place when a charging node is close enough to use some form of wireless power transmission technique [6,7,8]. As far as the wireless power transmission is concerned, [6] discusses theoretical and practical aspects of the far-field powering for low-power wireless sensors. Results for a prototype and design steps are also put forward in [6]. Special attention is devised for the project of the rectenna (rectifying antenna), which is the main element responsible for converting the electromagnetic incident wave energy into the energy used to charge the sensor nodes’ batteries.

### 1.1. Related Work

Much research effort has already been spent for improving the lifetime of WSNs, based for instance on routing protocols, transmission power control, network coding, MAC layer design, and autonomic approaches. Below, we provide a non-exhaustive list of the efforts and their main characteristics.

In [9], a framework for maximizing the network lifetime of a WSN is proposed exploring the sink node mobility under three constraints: prescribed delay tolerance regarding the transmission of the sensor information to the sink node, node energy consumption and data flow conservation. Nodes can temporarily store data until the mobile sink is in a better position for data exchange. The lifetime is a variable of the optimization problem that, when maximized, acts on minimizing the energy required per unit of time to transmit data at a certain bit rate in a multi-hop network topology. Comparisons with the static sink node scenario are also presented in [9].

The work in [10] focuses on finding the optimal sink node position with respect to relay nodes in order to prolong the WSN lifetime. An algorithm based on particle swarm optimization (PSO) is used to find the best position.

The authors of [11] propose a unified framework to analyze the network lifetime maximization problem in WSNs. A linear programming model jointly covers sink mobility and routing problems by constraining the sink to a finite number of locations. The authors apply the framework to a set of typical topological graphs, including linear, ring, grid and arbitrary topology. The paper reports lifetime improvements for mobile sinks when compared to static ones for different network sizes.

In [12], the authors introduce two linear programming models for data collection in a WSN with a controlled mobile base station and limited buffer capacity. The first model does not consider packet buffering, while the second model does. The paper is focused on comparing both scenarios considering static sensor nodes and a mobile sink. Sensors are randomly deployed, and the sink node can move according to a set of locations. Every sensor sends its data towards the sink either through multi-hop, or via direct communication if the sink is in the sensor’s vicinity.

In [13], a comparative study of WSN protocols is provided based on various network lifetime definitions, discussing the implications and applicability of the lifetime metrics. Several protocols are grouped into three classes according to similarities regarding lifetime definitions. Analyses are made based on the protocols that are representative to each class. Network-level simulation results are provided, showing numbers associated with the lifetime achieved by each protocol.

The authors of [14] deal with clustering algorithms where a number of sensors are distributed in a given area and a number of clusters with single cluster heads are formed. The positions of energy-harvesting nodes are adjusted for optimized network lifetime. The paper thus presents network lifetime improvements for clustered sensor networks.

The authors of [15] propose to exploit sensor spatial redundancy by defining subsets of sensors active in different time periods, allowing sensors to save energy when inactive. Two linear programming problems are presented to maximize network lifetime, which is defined in terms of the required coverage level: the first one, based on column generation, must run in a centralized way, whereas the second one is based on a heuristic algorithm aiming at a distributed implementation. The paper reports results to validate the model and to compare the adopted linear optimization problems.

A heterogeneous sensor network deployment strategy is proposed in [16]. Simulation is performed, primarily to measure to what extent memory and computation limiting can enhance network lifetime while attaining energy balancing. Network lifetime is defined as the time until the proportion of dead nodes exceeds a certain threshold, which is concluded to potentially fail in providing coverage, connectivity or both in a certain region. The proposed heuristic is called location-wise pre-determined heterogeneous node deployment (LPHND). It is compared with the relay node deployment with a Gaussian distribution (RNDGD) and the LPHND with placement error (LPHNDE) schemes.

The problem of controlling sink mobility in event-driven applications to achieve maximum network lifetime is the focus of [17]. The authors propose a convex optimization model inspired by the support vector regression technique to determine an optimal trajectory of the mobile sink. The network lifetime is defined as the elapsed time since the beginning of the network operation till the first active sensor node dies. Three optimization models are proposed and compared to a tabu search-based algorithm. The network is divided into zones that have one mobile sink. The paper reports results on the difference of average lifetime between the proposed convex models and the tabu search algorithm.

Approaches that act mainly on the energy spent during the task of communication among sensor nodes or between sensor nodes and sink nodes can be found in [5,17,18,19,20,21]. For example, transmission power control techniques aiming at managing energy consumption during communications are discussed in [18]. Some of these techniques use a single transmission power for the whole network; others apply local power management algorithms that do not involve MAC protocols. Additionally, time-division multiple access (TDMA) with collision avoidance and duty cycle strategies are also discussed in [18]. In [5], the authors propose a technique to mitigate the interference between WBANs using convex optimization in order to optimize the overall transmission power. Still in the context of optimization techniques, the authors of [22] consider the power consumption, throughput and delay as metrics related to the objective function of the optimization problem. It is shown in [20] that duty cycle control and network coding techniques can be integrated to utilize the network resources efficiently in the so-called bottleneck zone, which is the area around the sink node. A clustering algorithm based on the consumed energy in each time when a node becomes the cluster head is proposed in [19]. In [21], based on solid guidelines for lifetime improvement, a greedy algorithm that uses both the real-time channel state and the residual energy information is proposed for controlling the communications between the sensor nodes and the sink node.

### 1.2. Contributions and Structure of the Paper

This paper also deals with the problem of maximizing the lifetime of a mobile wireless sensor network and focuses on the energy spent during the task of communication among sensor nodes or between sensor nodes and sink nodes. It presents a novel convex optimization-based procedure (or method) that controls what we define as the communication activity levels of the sensor nodes, by dynamically assigning a number of time or time-frequency slots for communication with the sink nodes within a frame. The optimization concentrates on the communication task, since this is the most energy-consuming process of a sensor node [18]. Autonomous sensor node mobility is not considered here, since in this specific case large energy consumption would result from the mechanisms that produce mobility, as for instance in robots. Node movement can happen in a non-autonomous fashion, as for example when sensor nodes are attached to vehicles, animals, soldiers in the battle field, deployed in the sea, and so on. In all of these cases, the communication task will indeed consume most of the energy. In order to confer even more challenge to the problem solution besides mobility, it is assumed that the sensor network may adopt a wireless power transmission technique for battery recharging. The main attributes of the proposed method are:

- To the best of our knowledge, it differs from any previous one in the literature. The main novelty resides in the form of saving energy and in the formulation and solution of the corresponding optimization instance.
- It is flexible, allowing for the control of the periodicity in which the optimization tasks are performed, according to the sensor nodes’ mobility speeds: lower speeds require less frequent optimizations.
- It also allows for a flexible control over the trade-off between equalizing the sensor nodes’ energy consumptions to make them die altogether and minimize the wasted (not used) energy, or relaxing on the energy consumptions in favor of longer lifetimes by means of bursty transmissions followed by silent periods. When the equalization of energy consumptions is privileged, the communication with the sink node is frequent and avoids the waste of bandwidth caused by the bursty transmission mode when there is not enough data to send to the sink node. The frequent communication also avoids the storage of a high amount of sensory data until the next transmission is allowed in the bursty communication mode.
- It is simple, though fully consistent with well-known guidelines for the design of lifetime optimization algorithms reported in the literature.
- It performs closely to or better than a greedy algorithm recently proposed in the literature and strictly developed under the above-mentioned guidelines, with the advantage of not requiring the real-time channel state and residual energy information needed by the greedy solution.
- The reported results show that the optimized network can achieve substantially longer lifetimes when compared with the non-optimized one.

The remainder of the paper is organized as follows: Section 2 presents the problem statement and formulation. In Section 3, the corresponding solution via convex optimization is given. Numerical results and discussions concerning the performance of the proposed solution are reported in Section 4. Section 5 concludes the paper and gives some directions for future research.

## 2. Problem Statement and Formulation

**Figure 1:**
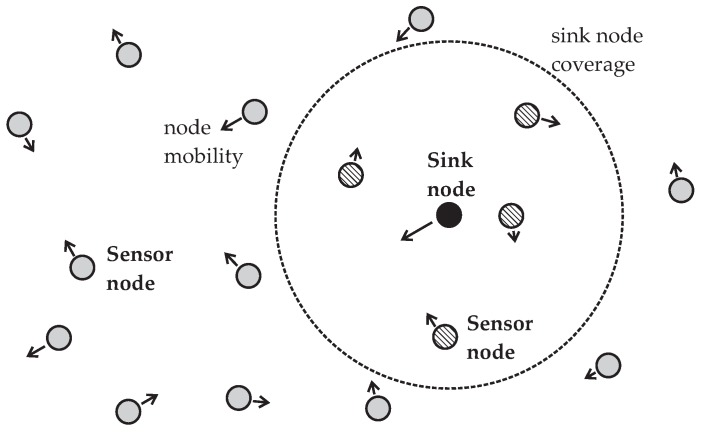
Topology adopted for the MWSN. The mobile sensor nodes inside the coverage area are those that can have direct wireless links to the mobile sink node. Others communicate with the sink node by means of multiple hops.

We consider an MWSN consisting of a number of sensor nodes whose communication tasks are controlled by a sink node or any other central node in a time-frequency-division basis. Figure 1 illustrates a possible topology adopted for the MWSN. The sensor nodes that can be directly controlled are those inside the sink node coverage area. Nonetheless, indirect control of all sensor nodes or a large portion of them can be achieved by means of multi-hop communication. Our lifetime optimization approach applies to both situations, but in the second one it must be combined with some suitable routing protocol.

When multiple hops are considered, two well-known network architectures can be defined, namely [21]: flat ad hoc and hierarchical ad hoc. Under the flat architecture, sensor nodes relay each other’s data to the sink node, whereas in the hierarchical architecture cluster heads send to the sink node the aggregated data from clustered sensor nodes. When direct communication with a mobile sink node is considered, it is said that the network is working under the SENMA (sensor network with mobile access) architecture.

Although many existing WSN protocols also handle routing to improve the network lifetime [11,23,24], here we consider the SENMA architecture so that the performance of our optimization approach can be decoupled from any routing protocol that could be working in parallel. Nonetheless, the potential lifetime improvements brought by our method can be assessed without loss of generality. A similar architecture is considered for instance in [25], and fits in applications such as intelligent transportation, environmental surveillance and wild life monitoring. The decoupling between the lifetime improvement method and routing is also considered in [21].

The sensor nodes, the sink node or both are allowed to move and, as a consequence, the distances from the sensor nodes to the sink node vary, thus varying the energy consumption during communication. The sensors use batteries that cannot be replaced, but in our model they are supposed to be sporadically charged by means of a wireless power transmission from a charge node (which can be the sink node itself). It is assumed that each sensor node spends most of its energy during communication events with the sink node. We associate the term activity level to the amount of time or time-frequency resources allocated to these events. Since the communication process is typically organized in frames (or superframes) and each frame is divided into time or time-frequency slots, then the activity levels will define the fraction of slots in a frame that each sensor is allowed to use for communication purposes. Due to the node mobility, the problem is to determine dynamically the optimum activity levels assigned to the sensor nodes so that the network lifetime is maximized. In this context, the proposed sensor node activity optimization model can be in principle applied to any wireless sensor network that employs slotted operation, especially those that employ a centralized manager to maintain the network schedule, e.g., WirelessHART [26], ISA100.11a [27,28] and IEEE 802.15.4e [29,30].

**Figure 2:**
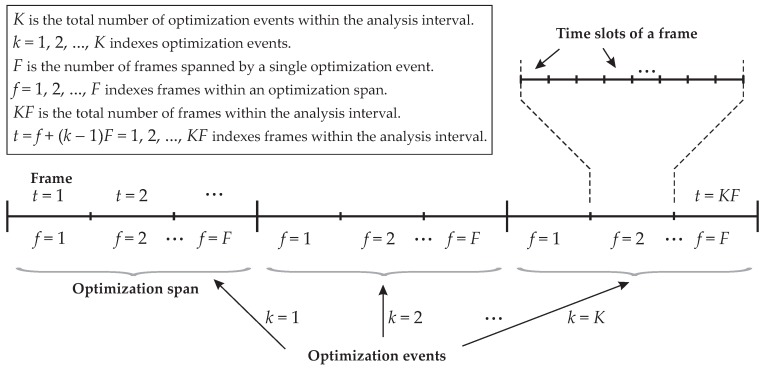
Time slotted approach for optimizing the activity levels of the mobile sensor nodes.

**Figure 3:**
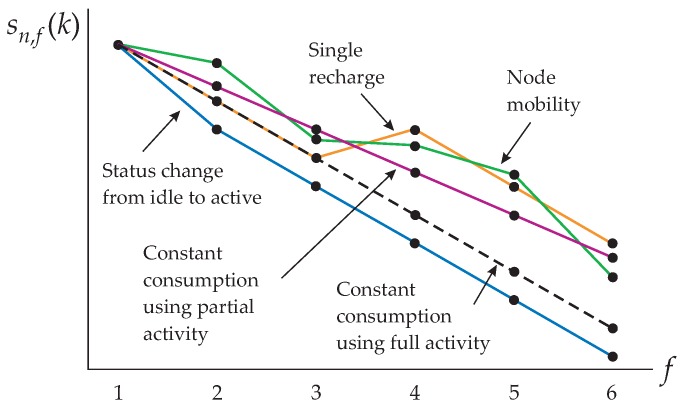
Pictorial representation of a net random energy consumption during the *k*-th block of F=5 frames for the *n*-th sensor node.

**Figure 4:**
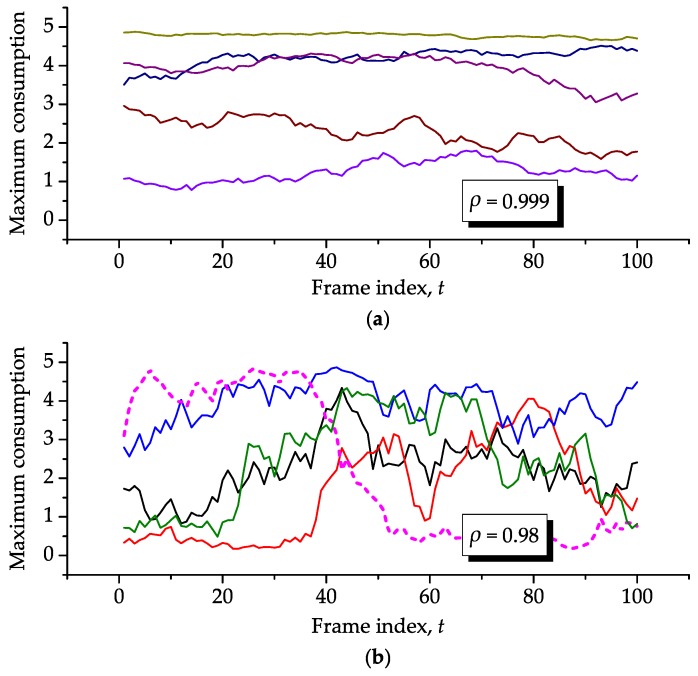
Pictorial representation of the maximum energy consumptions of five sensor nodes during 100 frames, for ρ=0.999 (**a**) and ρ=0.98 (**b**), for $B_{\min}=0.1$ and $B_{\max}=5$.

Before the presentation of the lifetime optimization method, some parameters, variables and the system model need to be defined:

- As depicted in the frame structure shown in Figure 2, an optimization event k=1,2,⋯,K spans *F* frames indexed by f=1,2,⋯,F, meaning that a lifetime optimization event acts on groups of *F* frames. The number of time or time-frequency slots in a frame is defined to accommodate the planned number of sensor nodes in the network. We call *F* the optimization span. Low mobility or fixed WSNs will demand less frequent optimization events, i.e., large *F*, whilst WSNs with high mobility nodes will need frequent optimizations, i.e., small *F* or even F=1.
- The value of KF is associated with the interval of analysis corresponding to KF frames indexed by t=f+(k-1)F=1,2,⋯,KF, and represents the time over which the network is in operation. In a real network, the parameter KF is not specified, in this case being determined by the network lifetime. The value of KF, though, can be determined by the network administrator so that the optimization ceases at a desired moment while the network is fully operative.
- The number of sensor nodes is denoted by *N*, and they are indexed by n=1,2,⋯,N. A single sink node is assumed.
- The energies in the sensor nodes’ batteries associated with the *k*-th optimization event, k=1,2,⋯,K, are given by the residual energy matrix $S\left(k\right)=\left[s_{1}\left(k\right),s_{2}\left(k\right),\cdots ,s_{F+1}\left(k\right)\right]\in R^{N\times (F+1)}$, with $s_{1}\left(1\right)$ being the vector with the initial energies stored in the batteries. After the action of the *k*-th optimization event, the residual energy values in the vector $s_{F+1}\left(k\right)$ become the initial energies for the next optimization event, that is $s_{1}\left(k+1\right)=s_{F+1}\left(k\right)$.
- The activity level matrix is defined by $X\left(k\right)=\left[x_{1}\left(k\right),x_{2}\left(k\right),\cdots ,x_{F}\left(k\right)\right]\in R^{N\times F}$, with $x_{f}\left(k\right)⪰\;0$ and $1^{T}x_{f}\left(k\right)=1$, where ⪰ represents component-wise inequality, 1 is the all-one *N*-dimensional vector, and the superscript T denotes transposition. This matrix contains the activity levels assigned by the *k*-th optimization event to all sensor nodes in the *f*-th frame within the optimization span. If, for instance, an element $X_{n,f}\left(k\right)$ of the activity level matrix is 0.1, this means that the *n*-th sensor node can occupy up to 10% of the slots during the *f*-th frame pertaining to the *k*-th group of *F* frames. The amount of energy consumed by the sensor nodes is proportional to the assigned activity levels and depends on the channel conditions between the sensor nodes and the sink node. These channels conditions, in turn, depend on the relative position of each sensor node with respect to the sink node, which is assumed to be unknown. This is important in practice, since channel estimation is a computationally costly process that contributes with the increase of the energy consumption and of the system complexity.
- If the maximum activity level of one is assigned to the sensor nodes, the energies that they are expected to spend during the transmissions of *F* frames are represented by the maximum consumption matrix $B\left(k\right)=\left[b_{1}\left(k\right),b_{2}\left(k\right),\cdots ,b_{F}\left(k\right)\right]\in R^{N\times F}$, with B(1) denoting the expected maximum consumption during the first *F* frames. Then, during the *k*-th block of *F* frames, the energies that are expected to be consumed by the sensor nodes are given by B(k)∘X(k), where the symbol ∘ denotes the Hadamard product (element-wise multiplication). The elements of B(k) in practice will depend on the sensor nodes’ characteristics and on the distances and channels between the sensor nodes and the sink node.
- It is assumed that the estimated consumption during the first *F* frames is constant, yielding $b_{1}\left(1\right)=b_{2}\left(1\right)=\cdots =b_{F}\left(1\right)$. This means that transmissions during the first frame are assumed to be done before the sensor nodes start moving. These first transmissions do not need to carry useful sensory information, being used to correctly update the energy consumption for the next optimization event. In fact, there is no reason for having $b_{1}\left(1\right)\neq b_{2}\left(1\right)\neq \cdots \neq b_{F}\left(1\right)$. This would be impractical, since it would demand the prediction of the nodes movement during the first *F* frames. Then, if the sensor nodes positions are known at the initial deployment of the network, $b_{1}\left(1\right)$ can be estimated by means of simple link budgets. The transmit powers considered in this estimation are those enough for producing the target error probability at the sink node (the calculations regarding this link budget are exemplified in Section 4.1.4). Alternatively, the elements of $b_{1}\left(1\right)$ can be, for instance, the energy consumptions calculated from an average expected distance between the sensor nodes and the sink node. The subsequent maximum consumption matrix is computed based on the energy consumed by the sensor nodes during the previous *F* frames, taking into account past battery recharges and mobility.
- The recharge matrix is defined as $R\left(k\right)=\left[r_{1}\left(k\right),r_{2}\left(k\right),\cdots ,r_{F}\left(k\right)\right]\in R^{N\times F}$, with $r_{f}\left(k\right)$ representing the amount of energy delivered to the sensor nodes’ batteries during recharge. In practice, the instants of recharge and the amount of charge transferred to the sensors’ batteries will depend on the energy harvesting method and the associated technology. For instance, recharge can occur very frequently if the energy harvesting is from a continuously-transmitted radio-frequency (RF) signal, opportunistically in the case of solar energy harvesting, or defined according to when and how often a recharging device passes through the network transmitting RF signals for the specific purpose of RF power delivery. As a case study, here we consider that recharge is achieved by means of RF energy transmission in the *f*-th frame of the *k*-th block of frames. In this case, it is reasonable to assume that R(k) is a sparse matrix, since in practice this type of recharge event is sporadic. This means that $r_{f}\left(k\right)=0$ for the majority of *f* and *k*, where 0 is the all-zero *N*-dimensional vector. We assume that recharge occurs at a single instant $t=t_{r}$ during the interval of analysis, meaning that the indexes of the recharge vector $r_{f}\left(k\right)$ will be the single pair of positive integers *f* and *k* that satisfy $t_{r}=f+\left(k-1\right)F$. We define the auxiliary parameter $r_{r}$, which is the fraction of the maximum stored energy $\max\{s_{1}\left(1\right)\}$ that limits the recharge energies, that is $r_{f}\left(k\right)⪯r_{r}\max\left\{s_{1}\left(1\right)\right\}$. To mimic possible different recharge energies applied to the sensor nodes’ batteries, we set the elements of $r_{f}\left(k\right)$ uniformly distributed in $\left[0,r_{r}\max\left\{s_{1}\left(1\right)\right\}\right]$. A more realistic model for RF energy transmission and recharge is by far more intricate and complex than the model just described, but the proposed simplification suffices for the purpose of lifetime analysis, without loss of generality.
- In practice, each row of the maximum consumption matrix B(k+1) that will be used as input to the (k+1)-th optimization event is formed at the sink node (or other central node) by the element-wise division between the actual energies consumed by the sensor nodes during the *k*-th block of *F* frames and the corresponding activity levels (recall that each element of the maximum consumption matrix contains the energy consumption of a sensor node when its activity level is one). The energy consumption comes from the residual energy and recharge level that can be measured by each sensor node and reported to the node where the optimization problem is solved to be applied to the next *F* frames. For now, assume that the sensor nodes fully use their activity levels assigned by the *k*-th optimization event. Then, the residual energies in their batteries at the end of the (f+1)-th frame are the residual energies at the end of the *f*-th frame minus the energy consumption, plus possible recharge, yielding
  (1) $$s_{f+1}\left(k\right)=s_{f}\left(k\right)-b_{f}\left(k\right)\circ x_{f}\left(k\right)+r_{f}\left(k\right).$$
  Then, the *f*-th column of B(k+1) can be computed according to
  (2) $$b_{f}\left(k+1\right)=\left[s_{f}\left(k\right)-s_{f+1}\left(k\right)+r_{f}\left(k\right)\right]\circ \frac{1}{x_{f}\left(k\right)},$$
  where $s_{f}\left(k\right)$ and $r_{f}\left(k\right)$, f=1,2,⋯,F, are the information on residual energies and recharge that is sent by the sensor nodes to the central node.
- However, it happens that the actual energy levels may wander due to the combined effects of node mobility, activity levels assigned and not fully used, and variations in the sensor node operation, for instance caused by state changes or by different processing needs depending on the sensed physical phenomena. Figure 3 illustrates possible evolutions of the actual residual energy $s_{n,f}\left(k\right)$ from the *f*-th to the (f+1)-th frame within an arbitrary block of F=5 frames, for the arbitrary *n*-th sensor node during the *k*-th block, assuming fixed activity levels and identical residual energies at f=1. From this figure, the random nature of the energy consumption of a sensor node is apparent, even if a fixed activity level is assigned to it.
- From above, each row of the maximum consumption matrix can be formed from correlated values of a random variable that will simulate variations in the residual energies as illustrated in Figure 3. The correlation level will determine the rate of variation in the energy consumptions from one frame to the next due to the previously-described combined effects. Specifically, let the auxiliary parameters $B_{\min}$ and $B_{\max}$ denote the minimum and maximum energy consumptions if the sensor node transmitter is on during the whole interval of a frame (which only happens if its activity level is one), when positioned at minimum and maximum distances from the sink node, respectively. Then, to mimic sensor nodes deployed at random positions, yielding different distances from the sink node and, thus, different consumptions, the elements of $b_{1}\left(1\right)=b_{2}\left(1\right)=\cdots =b_{F}\left(1\right)$ are assumed to be uniformly distributed in $\left[B_{\min},B_{\max}\right]$. A larger ratio $N/B_{\max}$ implies less sparse sensor nodes with respect to each other, i.e., a more dense network. The correlation coefficient between the energy consumptions in two consecutive frames is denoted by *ρ*. As an example, Figure 4 shows two realizations of the maximum consumptions of five sensor nodes under two values of *ρ*. The method adopted for generating the correlated uniform random variates shown in this figure is from the [31] (Chapter 7) and [32], assuming a triangular correlation function to guarantee the same correlation coefficient between the consumptions during any two neighbor frames. A low mobility network is represented in the graph (**a**) of this figure, whilst a relatively higher mobility is considered in the graph (**b**). In this last case, notice from the dashed line that the maximum consumption of a sensor node goes from $B_{\max}$ to $B_{\min}$ within 10 frames, which represents a quite large node speed in practice, despite the apparently high value of *ρ*.
- It is defined that the lifetime of a network is the time interval during which all sensor nodes are in full operation. In other words, the instant at which the first sensor node fails with high probability (does not work properly) or fails permanently (ends its operation or die) due to insufficient energy will determine the network lifetime. This definition has been adopted in several references, as for instance in [13,21,23,24,33,34,35,36,37]. The value of *t* at which the residual energy of any sensor node of the network becomes less than or equal to a given fraction of its maximum stored energy $\max\{s_{1}\left(1\right)\}$ is defined as the death instant, $t_{d}$, and the corresponding residual energy is defined and the death energy $s_{d}$. The death energy is a sensor-dependent parameter that relies on the characteristics of the battery and on the battery voltage level in which the sensor node starts to fail in accomplishing part or the totality of its functions. Examples of practical voltage levels related to failures can be found for instance in [37]. It is worth emphasizing that the failure-related voltage level (which could be called death voltage) is related to the death energy, though the conversion between theses quantities is not straightforward.

## 3. Problem Solution

A question is worth answering, before presenting the problem solution: what is expected from an effective network lifetime optimization process? According to the guidelines reported in [21], one must strike a balance between the minimization of the wasted energy, which is the unused residual energy at the network death moment, and the minimization of the energy spent to report the sensory information to the sink node. The authors of [21] complement their guidelines by indicating that this can be accomplished by exploring channel state information (CSI) and residual energy information (REI). However, one must recall that estimating the CSI would render more complex devices and additional energy consumption. In the approach described in what follows, non-real-time REI is explored, and the CSI is not, conferring to it a simpler implementation.

In the optimization strategy proposed in this paper, the energy balance is achieved by dynamically controlling the communication activity levels of the sensor nodes in a constant attempt to trade two goals: (i) make all sensor nodes consume the same amount of energy during communication, thus making them die together and yield the least possible wasted energy; (ii) prioritize longer lifetimes by relaxing on the equalization of energy consumptions to allow for bursty transmissions followed by silent periods. When the equalization of energy consumptions is privileged, the lifetime improvement is penalized. However, the communication with the sink node is more frequent, thus avoiding the waste of bandwidth caused by the bursty transmission mode when there is not enough data to send to the sink node at the very moment that it is allowed to transmit, yet avoiding the storage of a high amount of data until the next burst transmission is permitted in the bursty operation mode.

Then, in each optimization event, the objectives are to minimize the residual energies so that the unused energy is minimized and to minimize what we call the discounted residual energies. These discounted energies are formed by subtracting the potential maximum energy consumptions of the sensor nodes from their residual energies. As a consequence, higher residual energies combined with lower potential consumptions (better channels) will force the optimization to assign high activity levels, i.e., the corresponding sensor nodes will be granted burst-like transmissions. On the other hand, low residual energies combined with high potential consumptions (bad channels) will be associated with very low activity levels, i.e., the corresponding sensor nodes will be practically inactive while this situation remains.

Mathematically, in the *k*-th optimization event, a bi-criterion optimization problem is established in which the objective function is formed by the weighted sum of two objective functions, that is,

(3) $$f_{0}\left[s_{f}\left(k\right)\right]=w_{1}\max\left\{s_{f}\left(k\right)\right\}+w_{2}\max\left\{s_{f}\left(k\right)-b_{f}\left(k\right)\right\},f=1,2,\cdots ,F.$$

The variables of the optimization problem are the columns $s_{f}\left(k\right)$ of the residual energy matrix S(k) and the implicit activity levels, which are the columns $x_{f}\left(k\right)$ of the matrix X(k). The roles of the weights $w_{1}\geq 0$ and $w_{2}\geq 0$ are as follows: when $w_{1}/w_{2}>1$, the optimization acts in favor of equalized residual energies at the cost of reduced lifetime improvements; on the other hand, when $w_{2}/w_{1}>1$, the optimization tends to assign high activity levels to nodes with better channels and high residual energies, sometimes even disabling those under bad channels and having low residual energies, thus bringing the possibility of larger lifetime improvements. Assuming that the sensor nodes are deployed uniformly in the area of interest, when the number of sensor nodes is small, also small is the number of them in conditions to be assigned with low-consumption bursty transmissions (good channels and high residual energies), implying that $w_{2}/w_{1}>1$ will not bring advantage over $w_{1}/w_{2}>1$. In other terms, when *N* is small, say N<30, there is practically no difference between the lifetime improvements achieved by privileging equalized consumptions or by privileging bursty transmissions. In this case, total flexibility is given to the choice of the weights $w_{1}$ and $w_{2}$. When *N* is large, say N>80, the number of nodes in conditions to be assigned with low-consumption bursty transmissions increases, implying that $w_{2}/w_{1}>1$ will bring advantage over $w_{1}/w_{2}>1$. In this case, then, we must have $w_{1}=0$ and $w_{2}=1$, unless we can live with the lifetime penalty brought when the equalized consumptions are of more importance. For a moderate number of sensor nodes, say 30<N<80, we have found that a good trade-off is achieved with $w_{1}=1$ and $w_{2}=2$. Numerical results shown in Section 4 confirm these statements.

Notice that, from the perspective of a mathematical optimization problem, any pair of $w_{1}$ and $w_{2}$ will yield optimum solutions. By varying them, a Pareto optimum frontier [38] (p. 57) will be established, unveiling the typical losses and gains of a trade-off analysis.

At the *k*-th optimization event, the following constraints must be satisfied while minimizing the objective function defined in Equation (3):

- The activity levels must be non-negative, i.e., $x_{f}\left(k\right)⪰0$, f=1,2,⋯,F, meaning that the smallest number of slots assigned to a sensor node is zero.
- The activity levels assigned to the sensor nodes in a given frame must add to one, i.e., $1^{T}x_{f}\left(k\right)=\sum_{n=1}^{N}x_{n,f}\left(k\right)=1$, with $x_{n,f}\left(k\right)$ being the *n*-th element of $x_{f}\left(k\right)$. This constraint is consistent with the definition of the activity level as the assigned fraction of the slots in a frame. The effect of this constraint is the guarantee of not penalizing the throughput while the optimization attempts to minimize the residual and discounted residual energies in the objective function defined in Equation (3).
- The optimization computes the activity levels and the residual energies based on past maximum energy consumption information, that is, the residual energies available to the (f+1)-th frame, are the residual energies at the end of the *f*-th frame minus the energy consumptions during the *f*-th frame. Then, based on Equation (1), $s_{f+1}\left(k\right)=s_{f}\left(k\right)-b_{f}\left(k\right)\circ x_{f}\left(k\right)$, f=1,2,⋯,F. Notice that the maximum consumptions to be used as input data to the subsequent optimization event come from the actual information on past residual and recharge energies.

Putting it all together, for each optimization event *k* it must be solved the optimization problem

(4) $$\begin{array}{cc} \mathrm{minimize} & f_{0}\left[s_{f}\left(k\right)\right]=w_{1}\max\left\{s_{f}\left(k\right)\right\}+w_{2}\max\left\{s_{f}\left(k\right)-b_{f}\left(k\right)\right\},\;\;\;\;f=1,2,\cdots ,F\\ \mathrm{subject}\;\;\mathrm{to} & x_{f}\left(k\right)⪰0\\ & 1^{T}x_{f}\left(k\right)=1\\ & s_{f+1}\left(k\right)=s_{f}\left(k\right)-b_{f}\left(k\right)\circ x_{f}\left(k\right). \end{array}$$

This is a convex optimization problem, since the max function is convex [38] (p. 72), and the nonnegative weighted sum of convex functions is also convex [38] (p. 79). The optimization problem (4) can be easily converted into a linear optimization problem, usually referred to as a linear program (LP) [38] (p. 146), by means of its epigraph [38] (p. 134) form

(5) $$\begin{array}{cc} \mathrm{minimize} & z,\;\;\;\;f=1,2,\cdots ,F\\ \mathrm{subject}\;\;\mathrm{to} & w_{1}\max\left\{s_{f}\left(k\right)\right\}+w_{2}\max\left\{s_{f}\left(k\right)-b_{f}\left(k\right)\right\}-z\leq 0\\ & x_{f}\left(k\right)⪰0\\ & 1^{T}x_{f}\left(k\right)=1\\ & s_{f+1}\left(k\right)=s_{f}\left(k\right)-b_{f}\left(k\right)\circ x_{f}\left(k\right). \end{array}$$

It is worth mentioning that an LP is the most simple form among all convex optimization problems and that algorithms for its solution have matured to the point of providing extremely accurate results in a very short processing time. In the present case, even large values of *N* will not represent a limiting factor for accurate and fast solution of problem (4) or (5). Real-time convex optimization [39] approaches could be applied and embedded in digital signal processors if frame time requirements of the specific application become stringent. Nevertheless, the optimization problem (4) or (5) is meant to be solved for each *k* in a central node with more processing power than an ordinary sensor node.

The optimization variables obtained from the *k*-th optimization event are the activity level matrix $X\left(k\right)=[x_{1}\left(k\right),x_{2}\left(k\right),\cdots ,x_{F}\left(k\right)]$ to be assigned to the sensor nodes and the predicted (not necessarily actual) residual energy matrix $S\left(k\right)=[s_{1}\left(k\right),s_{2}\left(k\right),\cdots ,s_{F+1}\left(k\right)]$, k=1,2,⋯,K. The input parameters are the number of sensor nodes, *N*; the optimization span defined by *F*; the initial energy levels of the batteries, $s_{1}\left(1\right)$; and the estimated maximum consumed energies in the first *F* frames, $B\left(1\right)=[b_{1}\left(1\right),b_{2}\left(1\right),\cdots ,b_{F}\left(1\right)]$. The maximum consumption matrix that will be used as input to the event k+1 is updated using Equation (2), from the assigned activity levels and actual residual and recharge energies associated with the *k*-th block of *F* frames. There are two options for updating this matrix: each of its rows is computed by each sensor node and reported to the central node or the entire consumption matrix is computed at the central node from the residual and recharge information reported by the sensor nodes.

**Figure 5:**
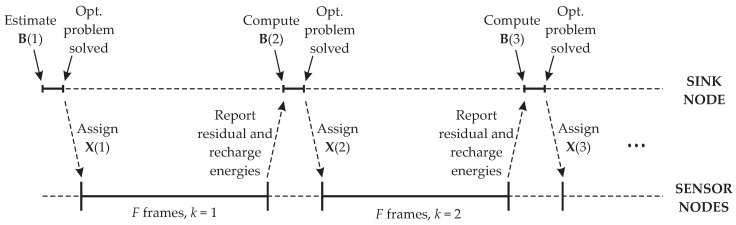
Time line illustrating the operation of Algorithm 1.

Algorithm 1 synthesizes the steps for optimizing the activity levels of the sensors nodes with the purpose of increasing the network lifetime. The last step of the algorithm is meant to indicate that the activity levels must be converted into a fraction of slots in a frame, this fraction being subsequently assigned to the sensor nodes by means of a proper scheduling or equivalent resource allocation algorithm. Figure 5 is meant to expose the time line of Algorithm 1, as well as the communication events between the sensor nodes and the sink nodes.

**Algorithm 1:** Optimization of the sensors’ activity levels. **Input**:  Number of sensor nodes, *N*,   Optimization span, *F*,   Initial energy levels of the batteries, $s_{1}\left(1\right)$,   Estimated consumed energies in the first *F* frames, B(1),  **for**
k=1,2,⋯ (up to the network death),   Solve the optimization problem (4) or (5) to determine X(k).   Compute matrix B(k+1) from sensor node information on actual residual energies and recharge.   Assign time or time-frequency slots to the sensor nodes according to the activity levels in X(k).  **end for**

## 4. Numerical Results

For all cases analyzed in this section, the optimization problem (4) was solved using the CVX, which is a system that works under the MATLAB environment for modeling and solving convex optimization problems [40]. The MATLAB-CVX code developed to generate the results shown hereafter is given in the Appendix.

For all results presented in this section, the initial energy in the sensor nodes’ batteries, the energy consumptions and sporadic battery recharge values are not actual ones, but scaled so as to anticipate the network death with respect to a real one, thus preventing the simulations of lasting prohibitively large intervals. Such a scaling has been also adopted for instance in [21], and though it changes the absolute lifetimes, it does not affect the conclusions regarding relative lifetime improvements or comparisons.

The results regarding the non-optimized network were obtained by setting identical equivalent activity levels $X_{n,f}^{\prime }\left(k\right)=1/N$ to all sensor nodes during the whole interval of analysis, with $X_{n,f}^{\prime }\left(k\right)$ being the element in the *n*-th row and *f*-th column of the equivalent activity level matrix $X^{\prime }\left(k\right)$ associated with the *k*-th block of *F* frames. Notice that these activity levels are nothing more than the average of the activity levels of all sensor nodes, which add up to one, during all optimization events up to the death instant of the network. As a consequence, the sensor nodes’ residual energies in the non-optimized network vary as determined by the their consumptions computed according to $X^{\prime }\left(k\right)B\left(k\right)$, with B(k) being the same maximum consumption matrix considered in the optimized network.

### 4.1. Influence of System Parameters

The effects of system parameters on the performance of the proposed lifetime optimization method are analyzed in this subsection. For the sake of a clear visualization of the results in the graphs, firstly a small number of sensor nodes is considered, i.e., N=10. Larger numbers of nodes are analyzed subsequently. Whenever a normalized energy value is called, it means that its value was normalized with respect to the maximum stored energy in the sensor node’s battery, i.e., $S_{\max}$. When it is stated that a single realization of random consumptions was adopted, it means that the maximum energy consumptions of the sensor nodes were generated for the whole interval of analysis according to the correlation coefficient *ρ* and the limits $B_{\min}$ and $B_{\max}$ and stored to be reused under different configurations of the system parameters. The death energy was set to 5% of $S_{\max}$. In practice, this relatively low percentage death energy corresponds to a high percentage of the nominal battery voltage associated with the death voltage [37,41]. For example, when the sensor’s battery voltage level is still at a high percentage of its nominal value, say 70%, the energy remaining in the battery is a low percentage of the maximum, say 5% or less [41].

#### 4.1.1. Effect of the Objective Function Weights

**Figure 6:**
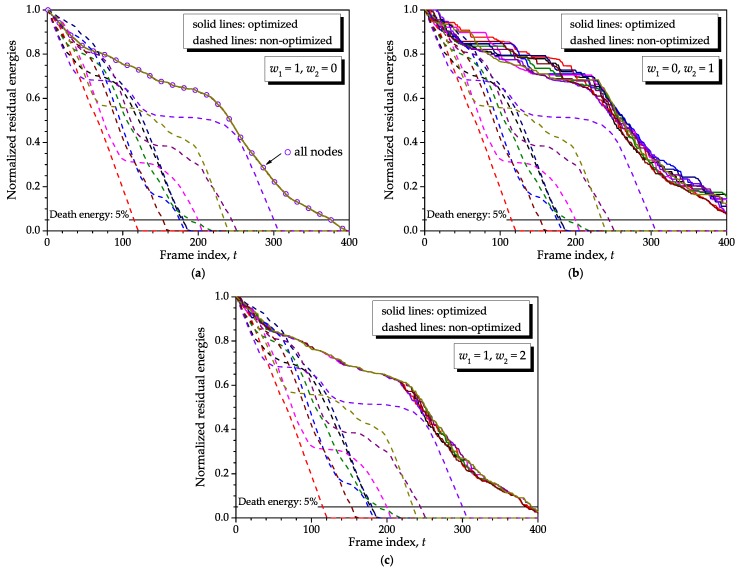
Residual energies obtained by the proposed optimization method for $\left(w_{1},w_{2}\right)=\left(1,0\right)$ (**a**); $\left(w_{1},w_{2}\right)=\left(0,1\right)$ (**b**); and $\left(w_{1},w_{2}\right)=\left(1,2\right)$ (**c**). The non-optimized network is considered in all graphs for reference.

Figure 6 shows the residual energies attained with the proposed optimization method under different weights in the objective function defined in Equation (3), namely $\left(w_{1},w_{2}\right)=\left(1,0\right)$ (**a**), $\left(w_{1},w_{2}\right)=\left(0,1\right)$ (**b**) and $\left(w_{1},w_{2}\right)=\left(1,2\right)$ (**c**). The non-optimized network is considered in all graphs for reference. We have set N=10, K=400, F=1, ρ=0.98, no recharge, $B_{\min}=0.001$, $B_{\max}=1$, and identical initial energies $s_{1}\left(1\right)=S_{\max}\times 1$, with $S_{\max}=10$. A single realization of the energy consumptions was adopted. In the majority of graphs from here, for the sake of clarity we intentionally did not use lines with symbols; only colors were used to identify different sensor nodes.

From the graph (**a**) of Figure 6, it can be inferred that all sensor nodes are allowed frequent communication with the sink node in a way that they spend approximately the same amount of energy as time elapses, that is, nodes will die altogether. Moreover, the unused residual energies at the network death moment, i.e., the wasted energy, is minimum due to the equalized consumptions. As will be shown ahead, for F>1, smaller *ρ* or both, the adoption of $\left(w_{1},w_{2}\right)=\left(1,0\right)$ would not equalize the consumptions precisely, yet reducing the network lifetime. This is due to the use of past information to optimize the current block of *F* frames: lower correlations combined with longer blocks represent past information less reliable with respect to the moment when it is used.

From the graph (**b**) of Figure 6, which considers $\left(w_{1},w_{2}\right)=\left(0,1\right)$, it can be observed that the optimization allows for burst-like transmissions combined with silent periods and continuous transmissions. The burst-like transmissions correspond to steep changes of the residual energies, whilst the intervals during which the residual energies do not change are associated with disabled (silent) nodes. Notice that this form of weighting achieves an increased network lifetime with respect to $\left(w_{1},w_{2}\right)=\left(1,0\right)$. As will be demonstrated later on in this paper, this effect is even more pronounced when the number of sensor nodes is larger.

In the graph (**c**) of Figure 6, the weights applied to the objective function were $\left(w_{1},w_{2}\right)=\left(1,2\right)$, resulting in a mixed situation in which consumptions are not perfectly equalized anymore and only some small burst-like transmissions and silent periods occur. No penalty and no improvement is observed in the network lifetime with respect to the one achieved when $\left(w_{1},w_{2}\right)=\left(1,0\right)$, which happens for a moderate to small number of sensor nodes. If *N* is large, both $\left(w_{1},w_{2}\right)=\left(1,0\right)$ and $\left(w_{1},w_{2}\right)=\left(1,2\right)$ would bring a lifetime penalty, which will be smaller for $\left(w_{1},w_{2}\right)=\left(1,2\right)$.

In all situations considered in Figure 6, the lifespan improvements were equal to or greater than 375/115≈3.27 times. Even larger lifetime records and, thus, lifetime improvements can be unveiled as the system parameters are chosen such that the network death occurs later, as for instance if the initial energy is increased while the consumptions are kept unchanged, if the consumptions are decreased for the same initial energy or if the number of sensor nodes is increased. This happens because the slopes associated with the residual energies in the optimized and non-optimized networks become more separated from each other as *t* increases. Then, in a real network in which the expected lifetimes are much higher than those simulated here, one might expect considerably larger lifetime improvements achieved with the proposed method. Another relevant conclusion obtained from Figure 6 refers to the fact that the energy levels tend to be more continuous when $\left(w_{1},w_{2}\right)=\left(1,0\right)$ and more step-like when $\left(w_{1},w_{2}\right)=\left(0,1\right)$. In the former case, it means that the sensor nodes are frequently having some opportunity to transmit their sensory data to the sink node, whereas in the later case some sensor nodes must remain silent during some intervals that can be relatively long.

**Figure 7:**
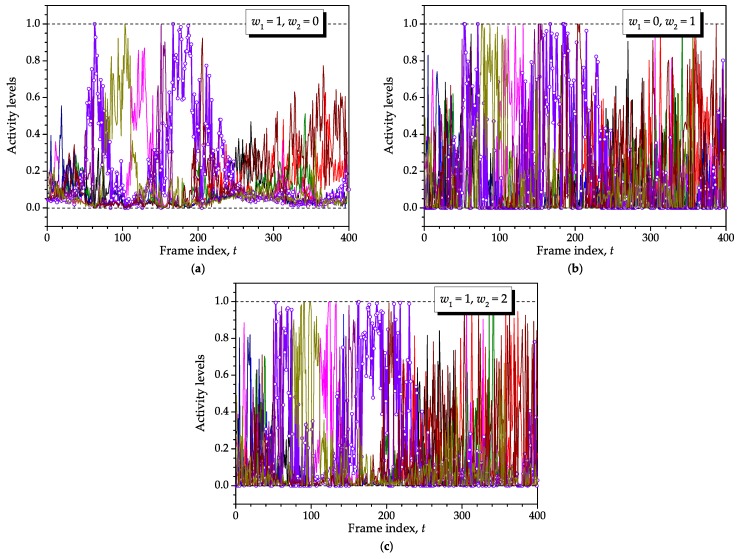
Activity levels assigned by the proposed optimization method respective to the situations considered in Figure 6, i.e., $\left(w_{1},w_{2}\right)=\left(1,0\right)$ (**a**); $\left(w_{1},w_{2}\right)=\left(0,1\right)$ (**b**); and $\left(w_{1},w_{2}\right)=\left(1,2\right)$ (**c**).

Figure 7 presents the activity levels assigned to the sensor nodes with respect to the residual energies shown in Figure 6. In general, those nodes that have low residual energy, are far away from the sink node or both are assigned smaller activity levels than those that are close to the sink node and have high residual energy. For example, observe that the sensor node identified with circles is assigned high activity levels around t=60 and t=180, meaning that it is under the combination of a good channel condition and enough residual energy around these intervals.

#### 4.1.2. Effect of the Optimization Span and Node Mobility

The optimization span *F* was defined in the proposed model to trade the frequency of the optimization events and the mobility speed of the sensor nodes, the sink node or both. Recall that a higher mobility speed can be simulated by reducing the correlation coefficient *ρ* between the energy consumptions in neighbor frames. The higher the mobility speed, the smaller must be the optimization span. The effects of these two parameters are analyzed simultaneously in this subsection. We consider F=5, F=10 and F=20 to complement the results already presented for F=1. We consider ρ=0.98 to represent a network with moderate-to-high mobility nodes and ρ≈1 to represent a fixed network (see Figure 4 and the related comments). In the former case, to keep consistency with previous results, we have used the same single realization of the consumptions throughout the KF frames. In the latter case, we have coined another realization of such consumptions with ρ=0.999999 and reused it. Equal initial energies $s_{1}\left(1\right)=S_{\max}\times 1$, with $S_{\max}=10$, $B_{\min}=0.001$, $B_{\max}=1$, and no recharge were assumed. Without loss of generality, we have chosen $\left(w_{1},w_{2}\right)=\left(1,0\right)$, N=10 and KF=400 so that the effects of interest are clearly seen in the corresponding graphs.

**Figure 8:**
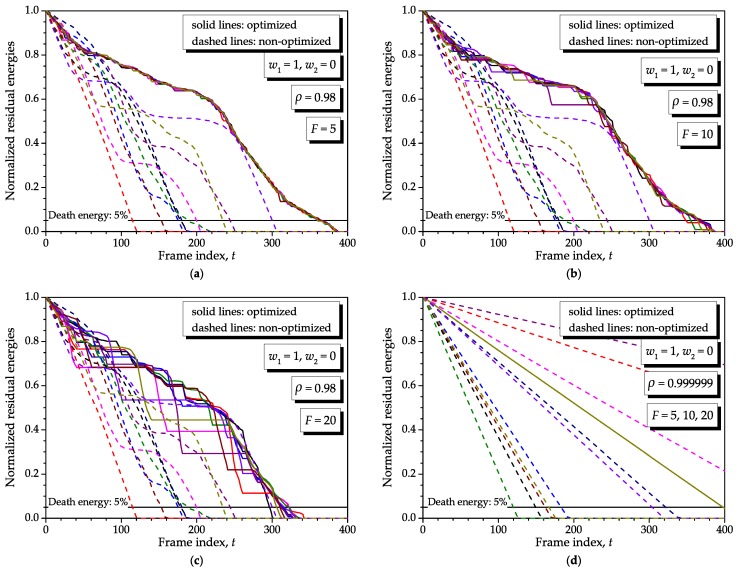
Residual energies obtained by the proposed optimization method with $\left(w_{1},w_{2}\right)=\left(1,0\right)$, for F=5 and ρ=0.98 (**a**); F=10 and ρ=0.98 (**b**); F=20 and ρ=0.98 (**c**); and F=5,10,20 and ρ=0.999999 (**d**). The non-optimized network is also considered for reference.

Figure 8 shows the results obtained by considering different optimization spans and correlation coefficients. Comparing the results for ρ=0.98, one can conclude that an increase in the optimization span produces an expected performance degradation, i.e., smaller lifetime improvements. This is due to the fact that past information on residual energies produces estimated consumptions progressively less correlated with the current ones as *F* increases. Comparing these results with the graph (**a**) of Figure 6, in which the death moment of the optimized network occurred around $t=t_{d}=375$, we have now death instants around $t_{d}=370$ for F=5, $t_{d}=350$ for F=10 and $t_{d}=300$ for F=20.

Now, observing the graph (**d**) of Figure 8, which considers fixed sensor nodes, it can be noticed that the lifetime does not change with *F*. Consequently, a few optimization events during the network operation would suffice to account for the variabilities in the energy consumption with respect to the one predicted by the optimization due to causes other than mobility, as for instance unused activity levels, sensor node state changes, different task-dependent processing burdens and the like.

#### 4.1.3. Effect of Recharge and Unequal Initial Energies

Without loss of generality, the effect of recharge and unequal initial energies can be separately analyzed from the single realization of the consumptions used before and an optimization span F=1. Again, we have used N=10, K=400, $B_{\min}=0.001$, $B_{\max}=1$ and ρ=0.98.

**Figure 9:**
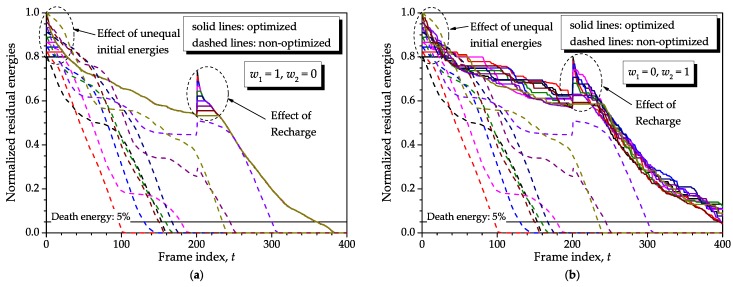
Residual energies obtained by the proposed optimization method for $\left(w_{1},w_{2}\right)=\left(1,0\right)$ (**a**) and $\left(w_{1},w_{2}\right)=\left(0,1\right)$ (**b**) with recharge and unequal initial energies. The non-optimized network is also considered for reference.

Figure 9 shows some results assuming normalized (with respect to $S_{\max}$) initial energies equally spaced in [0.8,1] and a single recharge at $t=t_{r}=200$, with normalized recharge values equally distributed in [0,0.2]. These equally-spaced energies were adopted to facilitate visualization in the graphs. From this figure, the expected lifetime reduction produced by initial energies smaller than the maximum can be noticed, combined with the expected lifetime improvement achieved due to recharge, no matter the weights of the objective function. Nonetheless, the most important observation comes from the equalization of the consumptions departing from unequal initial energies and the reestablishment of the normal operation of the optimization process after the disruption in the residual energies caused by recharge. Observe that the sensor nodes with less initial energies are kept silent over larger intervals compared to those with larger initial energies, up to the moment when all consumptions are equalized in the case of $\left(w_{1},w_{2}\right)=\left(1,0\right)$ or the characteristic operation is reestablished in the case of $\left(w_{1},w_{2}\right)=\left(0,1\right)$. A similar behavior occurs regarding the recharge.

**Figure 10:**
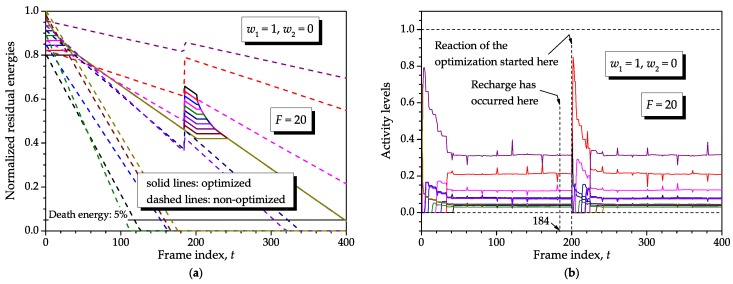
Residual energies (**a**) and activity levels (**b**) from the proposed optimization method for $\left(w_{1},w_{2}\right)=\left(1,0\right)$, with recharge at $t=t_{r}=184$ and unequal initial energies. The non-optimized network is also considered on the graph (**a**) for reference.

It is worth highlighting that the effect of recharge only starts to be taken into account by the optimization during the block of frames subsequent to the one in which the recharge event occurred. This is not evident in Figure 9 because F=1. In Figure 10, this specific character is demonstrated in terms of the residual energies and corresponding activity levels. To plot this figure, we have adopted F=20 and the same previously considered realization of the consumptions with ρ=0.999999. The set of initial energies and recharge values was the same as before, i.e., normalized initial energies and recharge values equally spaced in [0.8,1] and [0,0.2], respectively. For convenience, the recharge instant is now at $t=t_{r}=184$, that is it occurs sixteen frames before the beginning of the eleventh block of F=20 frames. From the graph (**a**) of Figure 10, a behavior similar to the one identified in Figure 9 can be noticed regarding the unequal initial energies. However, the effect of recharge is now different: notice that after recharge, the sensor nodes maintain their energy expenditures, up to t=200, as defined by the previous optimization event, that is the discharge rates are the same irrespective of the new energy levels in the sensor nodes’ batteries. Only at t=200 the optimization is aware of the recharge and controls the activity levels so that the consumptions start to be equalized again. The dynamics of the corresponding activity levels are shown in the graph (**b**) of Figure 10, from where the large variabilities associated with the adaptation to the unequal initial energies at the beginning of the analysis interval can be noticed and with the re-adaption due to recharge after t=200. The spikes at each integer multiple of F=20 in the activity levels are due to the compensation of small variations in the consumptions during the previous block. These variations exist since *ρ* is not exactly one and tend to diminish as ρ→1. As already mentioned, in practice such variations will always occur due to causes other than mobility; the spikes in the activity levels demonstrate the action of the optimization to compensate for these variations.

#### 4.1.4. Effect of the Node Density

As stated in Section 2, a larger ratio $N/B_{\max}$ implies less sparse sensor nodes with respect to each other, i.e., a more dense network. Then, the node density variation can be analyzed by varying *N* for a fixed $B_{\max}$ or by varying $B_{\max}$ for a fixed *N*. Both analyses are made in what follows, demonstrating the scalability of our method.

Before presenting the results, let us elaborate a little bit more on the node density, first defining it as the ratio between the number of nodes and the area where they are deployed. Let $d_{\min}$ and $d_{\max}$ be the minimum and maximum distances that a sensor node can be from the sink node, respectively. The minimum distance will define $B_{\min}$, and the maximum distance will define $B_{\max}$. Assuming that the sink node wireless coverage is circular and that a sensor node can be at any position within this circular area, then $d_{\min}=0$ and $d_{\max}$ is the radius of the coverage area. Thus, the node density, in nodes per square meter, can be defined as

(6) $$D=\frac{N}{\pi d_{\max}^{2}}.$$

In practice, the energy consumption will be proportional to the transmit power necessary for a target error rate in the received data at the sink node, which in turn will depend on the physical layer specifications. Assume that this target error rate is achieved if the received signal power at the sink node is $P_{\mathrm{target}}$. From the log-distance path loss model [42] (pp. 199–202),

(7) $$P_{\mathrm{target}}=\frac{P_{\mathrm{ref}}\left(d_{0}\right)}{{\left(d/d_{0}\right)}^{\eta }},$$

where $P_{\mathrm{ref}}\left(d_{0}\right)$ is a reference power at a close-in reference distance $d_{0}$ from the sensor node transmitter, *η* is the environment-dependent loss exponent and *d* is the distance of analysis. Typical values of *η* range from two in the free-space propagation scenario to four or even more in obstructed areas. The energy consumed by a sensor node at a distance *d* from the sink node will be proportional to $P_{\mathrm{ref}}\left(d_{0}\right)$, that is it will be proportional to ${\left(d/d_{0}\right)}^{\eta }P_{\mathrm{target}}$. Given that $P_{\mathrm{target}}$ is constant and assuming $d_{0}=1$ meter without loss of generality, then the energy consumption of a sensor node located at a distance *d* from the sink node will be proportional to $d^{\eta }$. More specifically,

(8) $$\begin{array}{c} B_{\min}=Cd_{\min}^{\eta },\\ B_{\max}=Cd_{\max}^{\eta }, \end{array}$$

where *C* is a hardware-dependent and battery-dependent proportionality constant that accounts for the proper conversion from transmit power into consumed energy. From this result, we see that $d_{\max}$ is proportional to $B_{\max}^{1/\eta }$. With this result in Equation (6), it can be concluded that

(9) $$D\propto \frac{N}{B_{\max}^{2/\eta }}.$$

Assuming η=2 for simplicity, then the network density can be multiplied by any factor, multiplying *N* or dividing $B_{\max}$ by this factor.

In Figure 8, $B_{\min}=0.001$, N=10 and $B_{\max}=1$ have been considered, yielding a node density D∝10. Obviously, in a real network, the actual density will be typically smaller than 10 nodes per square meter, meaning that the above proportionality is typically smaller than one. Moreover, one must recall that we have rescaled the energy values to anticipate the network death, which is consistent with such a high node density.

**Figure 11:**
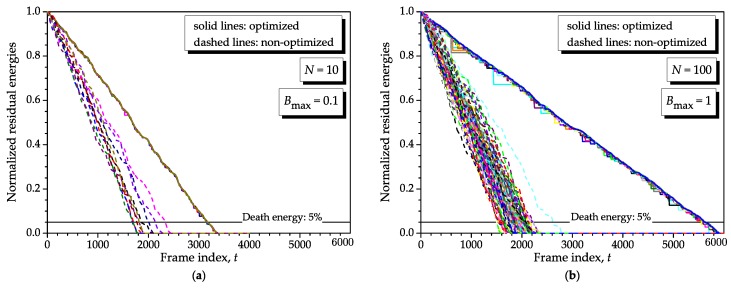
Residual energies obtained by the proposed optimization method for $\left(w_{1},w_{2}\right)=\left(1,0\right)$, F=10, $B_{\min}=0.001$ and ρ=0.98, with N=10 and $B_{\max}=0.1$ (**a**); and N=100 and $B_{\max}=1$ (**b**). The non-optimized network is also considered for reference.

Figure 11 shows the residual energies as produced by the proposed method with a 10-fold increase in the node density with respect to the one considered in the graph (**b**) of Figure 8, which has been chosen as representative for the present analysis. The graph (**a**) of Figure 11 considers $B_{\min}=0.001$, N=10 and $B_{\max}=0.1$, i.e., D∝100, whereas the graph (**b**) considers N=100 and $B_{\max}=1$, also yielding D∝100. From these results, the scalability of our method is demonstrated, and it is illustrated that the network lifetime increases as the number of nodes is increased or their energy consumptions are decreased. To plot the results in Figure 11, we have used two independent realizations of the sensor nodes consumptions during the corresponding interval of analysis.

### 4.2. Performance Comparisons

In this subsection we compare our lifetime optimization method with the so-called greedy algorithm from [21]. No recharge is assumed, as required by the greedy algorithm. In this algorithm, which is the best among those analyzed in that paper, the balance between minimizing the wasted energy and minimizing the energy spent to report the sensory information to the sink node is achieved by means of exploring both channel state information and residual energy information in real time. The net result is that each sensor node is allowed to transmit in bursts under the control of the sink node (or other central element), while the remaining nodes stay silent. The energy consumptions of the nodes thus vary in sharp steps, but in such a way that the overall energy is saved on average, prolonging the network life.

The greedy algorithm works as follows: the sensor node exclusively selected for transmission at a given moment is the one with maximum energy-efficiency index [21]

(10) $$\gamma_{n}=e_{n}-E_{r}\left(c_{n}\right),$$

where $e_{n}$ is the residual energy in the sensor node *n* at the beginning of a transmission and $E_{r}\left(c_{n}\right)$ is the required reporting energy as a function of the channel gain $c_{n}$ from the *n*-th sensor node to the sink node. Adapted to our notation, $E_{r}\left(c_{n}\right)$ corresponds to the element $B_{n,f}\left(k\right)$ of the maximum consumption matrix B(k) at each *k* and *f*. The residual energy $e_{n}$ starts with $s_{n,1}\left(1\right)$, i.e., the initial energy in the *n*-th sensor battery, and is updated for each *k* and *f* according to energy expenditure provided by the greedy algorithm, that is $e_{n}\leftarrow e_{n}-X_{n,f}\left(k\right)B_{n,f}\left(k\right)$, with $X_{n,f}\left(k\right)$ being the activity state, which is one for $n=\arg_{i}\max\gamma_{i}$, and zero otherwise. From this formulation, it is evident that the greedy algorithm uses current (real-time) channel state information $c_{n}$ and residual energy information $e_{n}$, whereas our method only uses past residual energy information. Notice that the term activity state is used here in the context of the greedy algorithm only to represent the *on* (activity state 1) and *off* (activity state 0) states of the sensor nodes’ transmitters as determined by the algorithm, thus establishing consistency with the term activity level defined in this paper.

**Figure 12:**
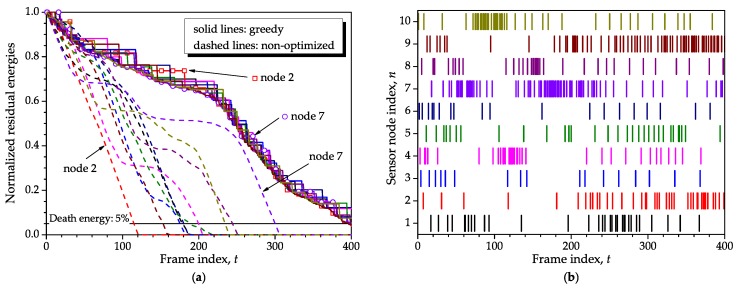
Residual energies (**a**) and sensor node activity states (**b**) produced by the greedy method of [21]. The vertical slashes on the graph (**b**) indicate *on* states, which are mutually exclusive for each *t*.

Figure 12 shows the residual energies (**a**) and corresponding sensor nodes’ activity states (**b**) obtained from the greedy algorithm of [21]. As in the case of Figure 6 and Figure 7, to plot Figure 12 we have also adopted K=400 optimization events, each one spanning F=1 frame; N=10 sensor nodes; identical initial energies $s_{1}\left(1\right)=10\times 1$; no recharge; death energy equal to 5% of the maximum; correlation coefficient of the consumptions given by ρ=0.98; and minimum and maximum consumptions $B_{\min}=0.01$ and $B_{\max}=1$. Again, a small number of sensor nodes was assumed for not polluting the graphs; higher values of *N* are considered later on in this section. The same single realization of the random consumption used to plot Figure 6 has been adopted here again.

From Figure 12, one can firstly see the burst-silent communication nature of the greedy algorithm, which happens independently of the number of sensor nodes. Observing the residual energies of the non-optimized network, it can be seen that the node numbered 2 has the steepest energy expenditure during the first 120 frames, implying that it is the farthest away from the sink node, on average, during the corresponding interval. As a consequence, this node was granted with fewer transmissions in this interval, as can be better noticed from the graph (**b**). On the other hand, sensor node 7, which is closer to the sink node on average, is enabled more times because of its advantageous position up to t=310. Moreover, notice from the nodes’ activity states that sensor node 7 is enabled more frequently around t=60 and t=180, which is consistent with the behavior of the proposed optimization method, as depicted in Figure 7.

Comparing Figure 6 and Figure 12, one can observe that the lifetime obtained from the proposed optimization method is comparable to or larger than the one achieved with the greedy algorithm. As will be demonstrated ahead, this is true for small *N* and any $w_{1}$ and $w_{2}$, whereas for large *N* the superiority of our method occurs mainly when $\left(w_{1},w_{2}\right)=\left(0,1\right)$. However, the unused residual energies at the network death moment, i.e., the wasted energy, tend to be higher for some sensor nodes in the greedy algorithm than in the proposed method. This higher wasted energy results from some sensor nodes being less frequently activated than others. From the graph (**a**) of Figure 12 and the graph (**b**) of Figure 6, it can be observed that the energy expenditures produced by the greedy algorithm resemble, to a certain extent, the ones provided by our method when $\left(w_{1},w_{2}\right)=\left(0,1\right)$. In other words, when $\left(w_{1},w_{2}\right)=\left(0,1\right)$ the proposed optimization allows for burst-like transmissions combined with silent periods and continuous transmissions, yielding a net result that can overcome the greedy algorithm, mainly for a higher number of sensor nodes. Larger lifetime records and, thus, larger lifetime improvements are unveiled in favor of the proposed method or the greedy algorithm, as the system parameters are chosen such that the network death occurs later, as for instance if the initial energy is increased while the consumptions are kept unchanged or the consumptions are decreased for the same initial energy.

Table 1 shows other results comparing the proposed lifetime optimization method and the greedy algorithm of [21], as obtained from 200 Monte Carlo events with random maximum consumption matrices. Given the myriad of possibilities in terms of system parameters, we have adopted those that fairly approximate the conditions imposed on our method and the greedy algorithm, mainly with respect to the efficient use of the information available. Specifically, recalling that the greedy algorithm operates under the assumption of knowing the current residual energy and channel state information, we have chosen ρ=0.98 (see Figure 4), and small optimizations spans F=1 and F=5. By doing so, the past residual energy information used by our method does not exhibit very low correlation with the present one. We have measured the lifetime improvements with N=10 and N=100 sensor nodes to assess scalability. Moreover, we have analyzed the two extreme situations in terms of the weights in the optimization problem (4): $\left(w_{1},w_{2}\right)=\left(0,1\right)$ is the configuration that produces an operation similar to the one achieved by the greedy algorithm, i.e., burst-like transmissions and larger lifetimes, whereas $\left(w_{1},w_{2}\right)=\left(1,0\right)$ privileges the equalized consumptions of the sensor nodes to the detriment of smaller lifetimes. Whenever possible, conclusions are drawn regarding the results that would be obtained if different setups were chosen.

A first important conclusion obtained from Table 1 is that larger lifetimes are achieved by the proposed method when $\left(w_{1},w_{2}\right)=\left(0,1\right)$, no matter the remaining parameters. The advantage over the greedy algorithm in this case is more pronounced when *N* is larger, with comparable standard deviations of the lifetimes. When *N* is small, the performance achieved by the proposed optimization method does not vary too much for different weights $w_{1}$ and $w_{2}$, giving to the network administrator full flexibility in terms of the balance between equalized consumptions and burst-like transmissions. As *N* is increased, the lifetime is reduced if the equalized consumptions are privileged, implying that the burst-like operation achieved with $\left(w_{1},w_{2}\right)=\left(0,1\right)$ must be chosen when longer lifetimes is the main concern. Notice, however, that when *N* is large, the adoption of $\left(w_{1},w_{2}\right)=\left(1,0\right)$ reduces the variations of the lifetimes, as demonstrated by the smaller standard deviations mainly in the case of N=100.

Still referring to Table 1, one can notice that the change from F=1 to F=5 did not produce significant performance degradation in our method, no matter the number of sensor nodes. Recall that, for F>1, past information on consumptions as used by our method become progressively more uncorrelated with the current ones, thus reducing the lifetime improvement efficiency. Then, if results with F>5 were presented in Table 1, yet larger performance degradations would be unveiled.

**Figure 13:**
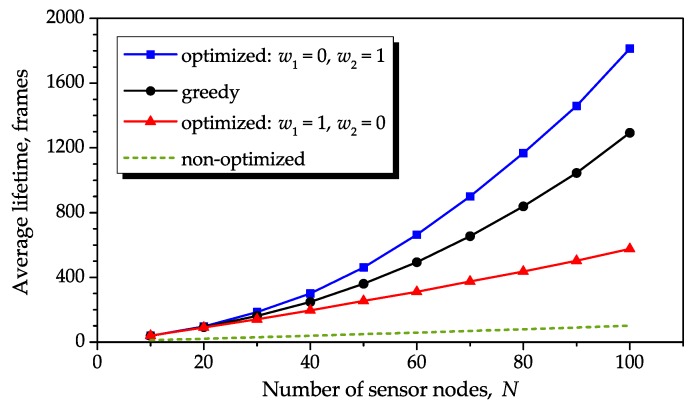
Average lifetimes achieved with the greedy algorithm of [21] and with the proposed optimization method for $\left(w_{1},w_{2}\right)=\left(1,0\right)$ and $\left(w_{1},w_{2}\right)=\left(0,1\right)$, under a variable number of sensor nodes, *N*. The average lifetime of the non-optimized network is also shown for reference.

As a final comparison, Figure 13 shows average lifetimes obtained from Monte Carlo simulations of the greedy algorithm and the proposed optimization method for $\left(w_{1},w_{2}\right)=\left(1,0\right)$ and $\left(w_{1},w_{2}\right)=\left(0,1\right)$, with a variable number of sensor nodes, *N*. The average lifetime of the non-optimized network is also shown for reference. To prevent simulations from having excessively large durations when *N* is large, the number of Monte Carlo events was varied linearly from 500 for N=10, to 50 for N=100, benefiting from the reduced lifetime variance as *N* increases (see Table 1). For a consistent comparison, we have adopted parameters from [21] adapted to our notation: equal initial energies $s_{1}\left(1\right)=S_{\max}\times 1$, with $S_{\max}=5$, and $B_{\min}=0.01$. The maximum energy consumption was empirically adjusted to $B_{\max}=5$ so that the performance of the greedy algorithm approximated the one reported in [21] (Figure 1) at the limits N=10 and N=100. From Figure 13, it can be concluded that our method attains comparable performances with respect to the greedy algorithm for a small number of sensor nodes. As this number increases, the performance of our method becomes worse than the greedy algorithm for $\left(w_{1},w_{2}\right)=\left(1,0\right)$ and superior for $\left(w_{1},w_{2}\right)=\left(0,1\right)$. The large lifetime improvements are also evident with respect to the non-optimized network, for any number of sensor nodes and any objective function weights.

From the results presented in this subsection, one can conclude that our method is capable of yielding performances comparable to those achieved by the greedy algorithm or even better ones. The stringent timing requirements regarding the channel state and residual energy information for the proper operation of the greedy algorithm give to our method a two-fold advantage: (i) a relaxed time requirement due to the use of past, instead of real-time information; (ii) the process of gathering energy consumption from the sensor nodes is by far less complex than estimating the channel state.

Another relevant advantage of our scheme when compared with the greedy algorithm of [21], especially when F>1, is that some sensor nodes with higher priority data can be prioritized to occupy the time or time-frequency slots before those nodes with lower priority data. This is due to the fact that the activity levels are determined to be applied in the subsequent block of *F* frames. On the other hand, in the greedy algorithm, a sensor node transmission must be done exactly when the channel is favorable to the enabled node, meaning that it cannot be prioritized.

### 4.3. Time Complexity

The effectiveness of an optimization method is usually measured by the computational complexity of its solution, which unveils the asymptotic processing time growth as the number of dimensions increases. However, the theoretical analysis of this complexity may pose stringent mathematical obstacles, which arise, for instance, due to the theoretical properties of the method, to the way it is implemented, to the choice of the programming language and to the parameter values [43,44]. This is particularly true in the case of the optimization problem (4): although it is convex, it has no closed form solution, and in our setup it was not solved using an application-specific code developed under a specific optimization algorithm. Instead, it was solved using the package CVX, which relies on the capabilities of the embedded solvers. Nevertheless, in this final subsection we present the results of an empirical complexity analysis by means of running time tests. The results of this analysis cannot be associated with practical processing times in absolute terms, but can give a rough idea of the relative processing time growths of the proposed lifetime improvement solution.

For computing the processing times, we have used the tic and toc MATLAB functions, which provide reliable elapsed time measurements. In order to reduce the effects of randomness, the measurements were averaged over 500 solutions of a single instance of the optimization problem (4), for the number of sensor nodes ranging from N=50 to N=1000 in steps of 50 and weighting factors $\left(w_{1},w_{2}\right)=\left(1,0\right)$, $\left(w_{1},w_{2}\right)=\left(0,1\right)$ and $\left(w_{1},w_{2}\right)=\left(1,1\right)$. The measurements were carried out in a Dell computer (Dell, Hortolândia, Brazil) with 2.2-GHz Intel Core i7-3632QM processors running the Windows 7 Professional operating system with Service Pack 1 and the 64-bit MATLAB R2010b (7.11.0.584) version.

For an optimization algorithm that exhibits polynomial time complexity in the number of sensor nodes, i.e., $O(N^{a})$, *a* is the power constant to be determined. We have used a power curve fitting function to find *a*. The results are summarized in Table 2, from where one can conclude that the largest time growth is $O(N^{1.44})$, which shows that our optimization method has a time complexity with a low power constant, meaning that it is tractable enough to be implemented in practice.

## 5. Conclusions and Future Work

This paper presented a novel method for increasing the lifetime of fixed or mobile wireless sensor networks. The method dynamically controls the so-called communication activity levels of the sensor nodes in a way that the fractions of time or time-frequency slots in a frame are optimally assigned to the sensor nodes to communicate with the sink node, at the same time saving energy.

The proposed method uses the solution of a convex optimization problem in which the objective function to be minimized is the weighted sum of the maximum residual energy and the maximum discounted residual energy, where the discount acts in favor of assigning higher activity levels to sensor nodes that have higher residual energies and better channels to the sink node. The net result is a balance between the minimization of wasted energy, the maintenance of throughput and the minimization of the energy spent for reporting sensory information to the sink node.

Since the optimization problem is convex, with the possibility of being transformed into a linear programming problem, mature and fast solvers available in practice can be efficiently applied. An empirical complexity analysis was carried out and unveiled that our optimization method has a time complexity $O(N^{1.44})$, meaning that it has potential for implementation in real networks.

Moreover, the proposed method is flexible enough to allow for controlling the frequency in which the optimization tasks are performed, according to the expected mobility of the nodes. Another merit with significant practical appeal of the proposed method is that it only uses past information on the residual energies and possible recharges reported by the sensor nodes, thus not demanding any real-time information.

Comparisons with a greedy algorithm that uses real-time channel state and residual energy information unveiled that the method proposed in this paper is capable of achieving comparable or even superior performances in most of the situations and that it is consistent with solid guidelines for the development of lifetime improvement strategies.

Some interesting investigations that represent opportunities for future work are:

1. The modeling of the communication task through queuing theory and its aggregation to the proposed optimization model is a natural first deployment. From this aggregation, one could analyze the influence of the optimization strategy in the data traffic associated with a realistic network model.
2. The adoption of a mobility model for the nodes is an important step towards the construction of an overall application-dependent lifetime analysis, as this model will have a great impact on the energy consumption pattern of the nodes.
3. The implementation of a real network operating under the proposed optimization method comes next, and here there is room for MAC and routing strategies that might benefit from the method and from possible connections with the Internet of Things (IoT), for instance in light of [45].
4. The application of the present optimization model to the design of a network level model in order to consider relaying or multi-hop communication is also an interesting point to be considered. This might improve even more the network lifetime by reducing the high energy consumption caused by the communication with distant central nodes in the centralized architecture, but would demand smarter sensor nodes to cope with the protocols associated with multi-hop communication.
5. The optimization strategy proposed here could be analyzed under different criteria for defining the network lifetime, taking for reference those definitions already considered for example in [13,33]. In this paper, it has been assumed that the network lifetime is the minimum lifetime over all nodes or, equivalently, it is determined by when the first sensor ceases operation. However, it is an application-specific task to determine when a network must be considered nonfunctional. It can be defined, for instance, when a percentage of sensors die or there is loss of coverage for some sensor nodes.
6. The modeling of the batteries’ charge and discharge is also a challenging investigation aiming at pushing the network simulation to a more realistic level. Two important aspects must not be forgotten in this modeling: the radio-frequency energy transmission (or other energy harvesting scheme) and the rate capacity effect [18], which is related to the discharge rate of the battery: drawing higher current than the rated value significantly reduces the battery lifetime and, thus, reduces the network lifetime. Graphene-based batteries [46] are promising devices that can dramatically influence these models and considerably push the network lifetime to unprecedented values.

## Figures and Tables

**Table 1:** Mean lifetimes (in frames), lifetime standard deviations and mean percentage lifetime improvements achieved with the proposed optimization method with different weights and with the greedy algorithm. These metrics are grouped between parenthesis as (optimized $w_{1}$ = 1 $w_{2}$ = 0; optimized $w_{1}$ = 0 $w_{2}$ = 1; greedy).

Parameters			Mean Lifetime, in Frames	Lifetime Std. Deviation	% Mean Lifetime Improvement
Death energy: 5% No recharge $B_{\min}=0.1$ $B_{\max}=1$ ρ=0.98	N=10 $s_{1}\left(1\right)=10$	K=400	(236; 242; 238)	(22; 25; 24)	(90; 98; 93)
		F=1			
		K=80	(230; 240; 236)	(22; 25; 23)	(87; 92; 90)
		F=5			
	N=100 $s_{1}\left(1\right)=1$	K=400	(243; 331; 289)	(8; 15; 13)	(127; 212; 173)
		F=1			
		K=80	(239; 315; 289)	(7; 18; 14)	(123; 197; 170)
		F=5			

**Table 2:** Average empirical time complexity of the proposed lifetime optimization method. The column on the right shows the growth order *a*.

Weights $w_{1},w_{2}$	Growth Order *a*
1,0	1.32
0,1	1.44
1,1	1.32

## Appendix A. MATLAB-CVX Source Code

This Appendix provides the MATLAB-CVX main code for solving the optimization problem (4), embedded in a MATLAB simulation that can be used for generating results like those presented throughout the paper. The outputs are: the run count for the simulations; the system parameters; the lifespans, mean lifespans and corresponding standard deviations; and mean lifetime improvements for the optimized network and for the network operating under the greedy algorithm. Graphs showing the associated activity levels and residual energies are also provided. Two other graphs are provided and may be enabled as desired.

Information on downloading and installing the CVX package, as well as the user guide and other related material can be found in [40].

%%%%%%%%%%%%%%%%%%%%%%%%%%%%%%%%%%%%%%%%%%%%%%%%%%%%%%%%%%%%%%%%%%%%%%%%%%%%%%%%%%%%%%%%%%%%
 
clear all; close all; clc; format long;
 
%%%%%%%%%%%%%%%%%%%%%%%%%%%%%%%%%%%%%%%%%%%%%%%%%%%%%%%%%%%%%%%%%%%%%%%%%%%%%%%%%%%%%%%%%%%%
%% SYSTEM PARAMETERS
%%%%%%%%%%%%%%%%%%%%%%%%%%%%%%%%%%%%%%%%%%%%%%%%%%%%%%%%%%%%%%%%%%%%%%%%%%%%%%%%%%%%%%%%%%%%
 
% Number of optimization events (or runs), indexed by k = 1,..., K:
K = 80;
 
% Number of frames spanned by each optimization event, indexed by f = 1,...,F:
F = 5;
 
% Number of sensor nodes, indexed by n = 1,...,N:
N = 10;
 
% Maximum initial stored energy of a sensor node. Reduce for higher N:
S_max = 10;
  
% Fraction of S_max such that the initial stored energies of the sensor nodes are
% U ~ [(1-Si_r)*S_max, S_max]. Si_r = 0 sets equal initial energies:
Si_r = 0.0;
  
% Death energy as a fraction of S_max:
Sd_r = 0.05;
 
% Recharge instant as a fraction of the total number of frames K*F:
tr_r = 0.46;
  
% Maximum recharge value as a fraction of S_max. Recharges are U ~ [0, r_r*S_max]:
r_r = 0.0;
  
% Minimum and maximum values for the maximum energy consumption matrix.
% Variations are U ~ [B_min, B_max]:
B_min = 0.001; B_max = 1;
  
% Correlation coefficient between the energy consumptions in one frame to the next.
% 0 <= rho < 1. Higher correlation means lower variations due to mobility speed,
% unused activity level, etc.:
rho = 0.98;
  
% Weights of the objetive functions (w1/w2 > 1 actcs in favor of equalized
% residual energies; w2/w1>1 tends to assign high activity levels to nodes with
% better channels AND high residual energies, sometimes disabling those under bad
% channels AND having low residual energies.
w1 = 1; w2 = 0;
  
%%%%%%%%%%%%%%%%%%%%%%%%%%%%%%%%%%%%%%%%%%%%%%%%%%%%%%%%%%%%%%%%%%%%%%%%%%%%%%%%%%%%%%%%%%%%
%% SET THE UPPER LIMIT OF repeat > 1 FOR REPEATED LIFETIME COMPUTATIONS
%%%%%%%%%%%%%%%%%%%%%%%%%%%%%%%%%%%%%%%%%%%%%%%%%%%%%%%%%%%%%%%%%%%%%%%%%%%%%%%%%%%%%%%%%%%%
  
for repeat = 1:1
  
%%%%%%%%%%%%%%%%%%%%%%%%%%%%%%%%%%%%%%%%%%%%%%%%%%%%%%%%%%%%%%%%%%%%%%%%%%%%%%%%%%%%%%%%%%%%
%% INITIAL COMPUTATIONS
%%%%%%%%%%%%%%%%%%%%%%%%%%%%%%%%%%%%%%%%%%%%%%%%%%%%%%%%%%%%%%%%%%%%%%%%%%%%%%%%%%%%%%%%%%%%
  
% Average expected lifetimes of the non-optimized network (just as information):
Min_lifetime_no = S_max/(B_max*(1/N))+1; Max_lifetime_no = S_max/(B_min*(1/N))+1;
  
% Correlation matrix of the actual maximum energy consumptions:
M_corr=zeros(K*F,K*F);
for i=1:K*F; for j=1:K*F; M_corr(i,j) = 1-(abs(i-j)*(1-rho)); end; end
  
% Correlation matrix for Gaussian-distributed consumptions:
M_corr = M_corr.*(M_corr>=0);
  
% Adjusting correlations for uniformly-distributed consumptions:
for i = 1:K*F; for j = max(K*F-1,i):K*F
if i ~= j; M_corr(i, j) = 2 * sin(pi * M_corr(i, j) / 6);
M_corr(j, i) = 2 * sin(pi * M_corr(j, i) / 6); end; end; end
  
% Inducing correlation:
Ch = chol(M_corr); G = randn(N,K*F); Br = G * Ch;
  
% Actual maximum consumption matrix with imposed limits:
Br = normcdf(Br)*(B_max-B_min) + B_min;
  
% Predicted constant maximum consumption during the first F frames:
for f = 1:F; B(:,f) = Br(:,1); end
  
% Initial energies in the sensor nodes's batteries for both the optimized and the
% non-optimized networks:
Si = S_max*ones(N,1).*(1-Si_r*rand(N,1)); Si_no = Si;
  
% Single recharge instant:
tr = floor(tr_r*K*F);
  
% Recharge matrix:
R = zeros(N,K*F); R(:,tr) = r_r*S_max*rand(N,1);
  
% Pre-allocation for storing the activity level and residual energy matrices for
% the optimized network:
Activity = []; Energy = [];
  
%%%%%%%%%%%%%%%%%%%%%%%%%%%%%%%%%%%%%%%%%%%%%%%%%%%%%%%%%%%%%%%%%%%%%%%%%%%%%%%%%%%%%%%%%%%%
%% OPTIMIZATION EVENTS (or runs)
%%%%%%%%%%%%%%%%%%%%%%%%%%%%%%%%%%%%%%%%%%%%%%%%%%%%%%%%%%%%%%%%%%%%%%%%%%%%%%%%%%%%%%%%%%%%
  
for k = 1:K
 
% Pre-allocation for energies and activity levels in each optimization event:
S=[Si]; X=[];
  
% Optimization solution:
for f=1:F
cvx_begin quiet
variables Xo(N,1) So(N,1)
subject to
Xo >= 0
sum(Xo) == 1
So == Si - B(:,f).*Xo
cvx_end
X=[X Xo]; Si=So;
end
  
% Actual residual energies:
for f = 1:F; S(:,f+1) = S(:,f) - Br(:,f+(k-1)*F).*X(:,f) + R(:,f+(k-1)*F);
end; S = S.*(S>=0);
% In practice, each row of matrix S and possible recharge values is the information
% sent from each sensor node to the sink node.
  
% Updating the maximum consumption matrix to be used in the next optimization
% event (it is equal to the present actual maximum consumption, which is obtained
% in practice from the sensor node information on the actual S and recharge R,
% using the assigned activity levels):
for f = 1:F; B(:,f) = (S(:,f)-S(:,f+1)+R(:,f+(k-1)*F))./X(:,f); end
% Notice that to compute consumption, recharge must be known. Otherwise recharge
% will act as if the consumption were smaller than the actual, or even negative
% (if recharge increases the residual energy above the previous one).
  
% Storing the activity levels and residual energies in the optimized network:
Activity = [Activity X]; Energy = [Energy S(:,(1:F))];
  
% Setting the initial energy for the next optimization event as the actual residual
% energy at the end of the current F frames:
Si = S(:,F+1);
  
end
  
%%%%%%%%%%%%%%%%%%%%%%%%%%%%%%%%%%%%%%%%%%%%%%%%%%%%%%%%%%%%%%%%%%%%%%%%%%%%%%%%%%%%%%%%%%%%
%% FINAL COMPUTATIONS
%%%%%%%%%%%%%%%%%%%%%%%%%%%%%%%%%%%%%%%%%%%%%%%%%%%%%%%%%%%%%%%%%%%%%%%%%%%%%%%%%%%%%%%%%%%%
  
% Death energy as a fraction Sd_r of S_max:
Sd = Sd_r*S_max;
  
% Death instants of the optimized network, and test for td outside the analyzed
% interval:
TD = ceil(find(Energy <= Sd, 1)/N);
if isempty(TD) == 1; TD = K*F;
errordlg(’Death instant beyond K*F. Re-define system parameters.’, ’WARNING’);
end; td(repeat) = TD;
  
% Activity levels for the non-optimized network:
x_no = ones(N,1)*(1/N);
  
% Pre-allocation for residual energies in the non-optimized and greedy networks:
Energy_no = [Si_no]; Energy_greedy = [Si_no];
  
% Evolution of the residual energies in the non-optimized and greedy networks:
for t = 1:K*F
Energy_no(:,t+1) = Energy_no(:,t) - Br(:,t) .* x_no + R(:,t);
g_greedy = Energy_greedy(:,t)+R(:,t)-Br(:,t); % Greedy’s energy efficiency index.
x_greedy(:,t) = g_greedy >= max(g_greedy); % Greedy "activity levels" (0 or 1).
Energy_greedy(:,t+1) = Energy_greedy(:,t) - Br(:,t).* x_greedy(:,t) + R(:,t);
end
  
% Discarding the last useless residual energies at t = K*F+1:
Energy_greedy = Energy_greedy(:,(1:K*F)); Energy_no = Energy_no(:,(1:K*F));
  
% Death instants of the non-optimized network:
td_no(repeat) = ceil(find(Energy_no<=Sd,1)/N);
  
% Death instants of the greedy network and test for td_greedy outside the analyzed
% interval:
TD_greedy = ceil(find(Energy_greedy<=Sd,1)/N);
if isempty(TD_greedy) == 1; TD_greedy = K*F;
errordlg(’Death instant beyond K*F. Re-define system parameters.’, ’WARNING’);
end; td_greedy(repeat) = TD_greedy;
  
Energy_no = Energy_no.*(Energy_no>=0); % Zeroing negative energies.
  
Energy_greedy = Energy_greedy.*(Energy_greedy>=0); % Zeroing negative energies.
  
%%%%%%%%%%%%%%%%%%%%%%%%%%%%%%%%%%%%%%%%%%%%%%%%%%%%%%%%%%%%%%%%%%%%%%%%%%%%%%%%%%%%%%%%%%%%
%% OUTPUT DATA AND GRAPHS
%%%%%%%%%%%%%%%%%%%%%%%%%%%%%%%%%%%%%%%%%%%%%%%%%%%%%%%%%%%%%%%%%%%%%%%%%%%%%%%%%%%%%%%%%%%%
  
figure(1); plot(Activity’); hold on; plot(sum(Activity/N),’y’,’lineWidth’,5);
legend(’optimized: solid lines’,’Average: thick line’,’Location’,’Best’);
axis([1 K*F 0 1.02*max(max(Activity))]); xlabel(’Frame index’);
ylabel(’Activity levels’); title(’Proposed optimization method’); hold off;
  
figure(2); plot(Energy’,’lineWidth’,2); hold on; plot(Energy_no’,’--’);
plot(td,Sd,’ok’,’lineWidth’,5); plot(td_no,Sd,’or’,’lineWidth’,5); grid on;
axis([1 K*F 0 S_max ]); xlabel(’Frame index’); ylabel(’Residual energies’);
legend(’optimized: solid lines’, ’non-optimized: dashed lines’,’Location’,’Best’);
title(’Proposed optimization method’);
hold off;
  
figure(3); plot(x_greedy’); hold on; plot(x_no’,’--’,’lineWidth’,2);
axis([1 K*F 0 1.02*max(max(x_greedy))]);
title(’Greedy algorithm’);
xlabel(’Frame index’); ylabel(’Activity levels’); hold off;
  
figure(4); plot(Energy_greedy’,’lineWidth’,2); hold on; plot(Energy_no’,’--’);
plot(td_greedy,Sd,’ok’,’lineWidth’,5); plot(td_no,Sd,’or’,’lineWidth’,5);grid on;
axis([1 K*F 0 S_max ]); xlabel(’Frame index’); ylabel(’Residual energies’);
legend(’Greedy: solid lines’, ’non-optimized: dashed lines’,’Location’,’Best’);
title(’Greedy algorithm’);
hold off;
  
% figure(5)
% Xg = x_greedy’; Xo = Activity’;
% Avg_X_greedy = sum(Xg(1:td_greedy(repeat),:))/(td_greedy(repeat));
% Avg_X = sum(Xo(1:td(repeat),:))/(td(repeat));
% bar(Avg_X_greedy,0.8); hold on; bar(Avg_X,0.5,’y’);
% legend(’greedy’, ’optimized’,’Location’,’Best’); xlabel(’Sensor node index’);
% ylabel(’Average activity over the lifespan’); hold off;
  
% figure(6); plot(Br(1:5,:)’,’Linewidth’,3);
% xlabel(’Frame index’); ylabel(’Maximum consumption of 5 sensor nodes’); hold off;
  
disp([’Optimized lifespan:        ’, num2str(td)]);
disp([’Greedy optimized lifespan: ’, num2str(td_greedy)]);
disp([’Run count: ’, num2str(repeat)]);
disp([’System parameters: ’,’K=’,num2str(K),’ F=’,num2str(F),’ N=’,num2str(N),
’ S_max=’,num2str(S_max),’ Si_r=’,num2str(Si_r),’ Sd_r=’,num2str(Sd_r),
’ tr_r=’,num2str(tr_r),’ r_r=’,num2str(r_r),’ B_min=’,num2str(B_min),’ B_max=’,
num2str(B_max),’ rho=’,num2str(rho),’ w1=’,num2str(w1),’ w2=’,num2str(w2)]);
disp([’Optimized mean lifespan:               ’, num2str(sum(td)/sum(td>0))]);
disp([’Greedy mean lifespan:     ’, num2str(sum(td_greedy)/sum(td_greedy>0))]);
disp([’Optimized lifespan standard deviation: ’, num2str(sqrt(var(td)))]);
disp([’Greedy lifespan standard deviation:    ’, num2str(sqrt(var(td_greedy)))]);
disp([’Optimized mean lifespan improvement:
’, num2str(100*(sum(td./td_no)/sum(td>0)-1)),’ %’]);
disp([’Greedy mean lifespan improvement:
’, num2str(100*(sum(td_greedy./td_no)/sum(td>0)-1)),’ %’]);
  
end
%%%%%%%%%%%%%%%%%%%%%%%%%%%%%%%%%%%%%%%%%%%%%%%%%%%%%%%%%%%%%%%%%%%%%%%%%%%%%%%%%%%%%%%%%%%%

## References

[1] Al-Jemeli M., Hussin F.A. (2015). An Energy Efficient Cross-Layer Network Operation Model for IEEE 802.15.4-Based Mobile Wireless Sensor Networks. IEEE Sens. J..

[2] Munir S., Ren B., Jiao W., Wang B., Xie D., Ma J. Mobile Wireless Sensor Network: Architecture and Enabling Technologies for Ubiquitous Computing. Proceedings of the 21st International Conference on Advanced Information Networking and Applications Workshops (AINAW ’07).

[3] Mitola J., Maguire G.Q. (1999). Cognitive radio: Making software radios more personal. IEEE Pers. Commun. Mag..

[4] Qu Z., Xu Y., Yin S. A novel clustering-based spectrum sensing in cognitive radio wireless sensor networks. Proceedings of the 3rd IEEE International Conference on Cloud Computing and Intelligence Systems (CCIS).

[5] Zhang Z., Huang J., Wang H., Fang H. Power control and localization of wireless body area networks using semidefinite programming. Proceedings of the 2nd International Symposium on Future Information and Communication Technologies for Ubiquitous HealthCare (Ubi-HealthTech).

[6] Popovic Z., Falkenstein E., Costinett D., Zane R. (2013). Low-Power Far-Field Wireless Powering for Wireless Sensors. Proc. IEEE.

[7] Li J.W. Wireless power transmission: State-of-the-arts in technologies and potential applications (invited paper). Proceedings of the 2011 Asia-Pacific Microwave Conference Proceedings (APMC).

[8] Mascarenas D.L., Flynn E.B., Todd M.D., Overly T.G., Farinholt K.M., Park G., Farrar C.R. (2010). Experimental studies of using wireless energy transmission for powering embedded sensor nodes. J. Sound Vib..

[9] Yun Y., Xia Y. (2010). Maximizing the Lifetime of Wireless Sensor Networks with Mobile Sink in Delay-Tolerant Applications. IEEE Trans. Mob. Comput..

[10] Rahman M.N., Matin M.A. (2011). Efficient algorithm for prolonging network lifetime of wireless sensor networks. Tsinghua Sci. Technol..

[11] Luo J., Hubaux J. (2010). Joint Sink Mobility and Routing to Maximize the Lifetime of Wireless Sensor Networks: The Case of Constrained Mobility. IEEE/ACM Trans. Netw..

[12] Rault T., Bouabdallah A., Challal Y. WSN Lifetime Optimization through Controlled Sink Mobility and Packet Bufferization. Proceedings of the 5th Global Information Infrastructure Symposium.

[13] Mak N.H., Seah W.K.G. How Long is the Lifetime of a Wireless Sensor Network?. Proceedings of the 2009 International Conference on Advanced Information Networking and Applications.

[14] Zhang P., Xiao G., Tan H.P. (2013). Clustering algorithms for maximizing the lifetime of wireless sensor networks with energy-harvesting sensors. Comput. Netw..

[15] Alfieri A., Bianco A., Brandimarte P., Chiasserini C. (2007). Maximizing System Lifetime in Wireless Sensor Networks. Eur. J. Oper. Res..

[16] Ghosal A., Halder S. (2016). Lifespan Prolonging Location-Wise Predetermined Deployment Strategy for Visual Sensor Networks. J. Netw. Comput. Appl..

[17] Tashtarian F., Hossein Yaghmaee Moghaddam M., Sohraby K., Effati S. (2015). On Maximizing the Lifetime of Wireless Sensor Networks in Event-Driven Applications With Mobile Sinks. IEEE Trans. Veh. Technol..

[18] Pinto A.R., Poehls L.B., Montez C., Vargas F. (2012). Power Optimization for Wireless Sensor Networks. Wireless Sensor Networks—Technology and Applications.

[19] Luo F., Jiang C., Zhang H., Wang X., Zhang L., Ren Y. Node Energy Consumption Analysis in Wireless Sensor Networks. Proceedings of the IEEE 80th Vehicular Technology Conference (VTC Fall).

[20] Rout R., Ghosh S. (2013). Enhancement of Lifetime using Duty Cycle and Network Coding in Wireless Sensor Networks. IEEE Trans. Wirel. Commun..

[21] Chen Y., Zhao Q. (2005). On the lifetime of wireless sensor networks. IEEE Commun. Lett..

[22] Munir A., Gordon-Ross A. (2010). Optimization Approaches in Wireless Sensor Networks. Sustainable Wireless Sensor Networks.

[23] Chang J.H., Tassiulas L. (2004). Maximum lifetime routing in wireless sensor networks. IEEE/ACM Trans. Netw..

[24] Chang J.H., Tassiulas L. Energy conserving routing in wireless ad-hoc networks. Proceedings of the INFOCOM 2000 Nineteenth Annual Joint Conference of the IEEE Computer and Communications Societies.

[25] Mehrabi A., Kim K. (2016). Maximizing Data Collection Throughput on a Path in Energy Harvesting Sensor Networks Using a Mobile Sink. IEEE Trans. Mobile Comput..

[26] HCF (2008). WirelessHART Specification 7.5: TDMA Data-Link Layer.

[27] The International Society of Automation (ISA) (2011). ISA-100.11a-2011: Wireless Systems for Industrial Automation: Process Control and Related Applications.

[28] Petersen S., Carlsen S. (2011). WirelessHART Versus ISA100.11a: The Format War Hits the Factory Floor. IEEE Ind. Electron. Mag..

[29] IEEE (2012). 802.15.4e-2012: IEEE Standard for Local and Metropolitan Area Networks—Part 15.4: Low-Rate Wireless Personal Area Networks (LR-WPANs) Amendment 1: MAC Sublayer.

[30] Martinez B., Vilajosana X., Chraim F., Vilajosana I., Pister K.S.J. (2015). When Scavengers Meet Industrial Wireless. IEEE Trans. Ind. Electron..

[31] Gilli M., Maringer D., Schumann E. (2011). Numerical Methods and Optimization in Finance.

[32] Schumann E. Generating Correlated Uniform Variates. http://comisef.wikidot.com/tutorial:correlateduniformvariates/.

[33] Dietrich I., Dressler F. (2009). On the Lifetime of Wireless Sensor Networks. ACM Trans. Sens. Netw..

[34] Cheng Z., Perillo M., Heinzelman W. (2008). General Network Lifetime and Cost Models for Evaluating Sensor Network Deployment Strategies. IEEE Trans. Mob. Comput..

[35] Ren J., Zhang Y., Zhang K., Liu A., Chen J., Shen X. (2016). Lifetime and Energy Hole Evolution Analysis in Data-Gathering Wireless Sensor Networks. IEEE Trans. Ind. Inform..

[36] Xiang M., Shi W., Jiang C., Zhang Y. (2010). Energy efficient clustering algorithm for maximizing lifetime of wireless sensor networks. AEU Int. J. Electron. Commun..

[37] Damaso A., Rosa N., Maciel P. (2014). Reliability of Wireless Sensor Networks. Sensors.

[38] Boyd S., Vandenberghe L. (2004). Convex Optimization.

[39] Mattingley J., Boyd S. (2010). Real-Time Convex Optimization in Signal Processing. IEEE Signal Process. Mag..

[40] Grant M., Boyd S. CVX: MATLAB Software for Disciplined Convex Programming, Version 2.1, Build 1110. http://cvxr.com/cvx.

[41] Simpson C. (2011). Characteristics of Rechargeable Batteries.

[42] Guimarães D.A. (2009). Digital Transmission: A Simulation-Aided Introduction with VisSim/Comm.

[43] Drori Y. (2014). Contributions to the Complexity Analysis of Optimization Algorithms. Ph.D. Thesis.

[44] Espinoza F.J.F. (2012). A New Interpolation Approach for Linearly Constrained Convex Optimization. Master’s Thesis.

[45] Hellbruck H., Pagel M., Kroller A., Bimschas D., Pfisterer D., Fischer S. Using and operating wireless sensor network testbeds with WISEBED. Proceedings of the 10th IFIP Annual Mediterranean Ad Hoc Networking Workshop (Med-Hoc-Net).

[46] El-Kady M., Kaner R. (2015). Introducing the micro-super-capacitor laser-etched graphene brings Moore’s law to energy storage. IEEE Spectr..

